# Boosting Nitrogen Reduction to Ammonia on FeN_4_ Sites by Atomic Spin Regulation

**DOI:** 10.1002/advs.202102915

**Published:** 2021-09-02

**Authors:** Yajin Wang, Wenzheng Cheng, Pengfei Yuan, Gege Yang, Shichun Mu, Jialin Liang, Huicong Xia, Kai Guo, Mengli Liu, Shuyan Zhao, Gan Qu, Bang‐An Lu, Yongfeng Hu, Jinsong Hu, Jia‐Nan Zhang

**Affiliations:** ^1^ College of Materials Science and Engineering Zhengzhou University Zhengzhou 450001 P. R. China; ^2^ International Joint Research Laboratory for Quantum Functional Materials of Henan Province and School of Physics and Microelectronics Zhengzhou University Zhengzhou 450001 P. R. China; ^3^ State Key Laboratory of Advanced Technology for Materials Synthesis and Processing Wuhan University of Technology Wuhan 430070 P. R. China; ^4^ Foshan Xianhu Laboratory of the Advanced Energy Science and Technology Guangdong Laboratory Xianhu Hydrogen Valley Foshan 528200 China; ^5^ Canadian Light Source 44 Innovation Boulevard Saskatoon Saskatoon SK S7N 2V3 Canada; ^6^ Beijing National Laboratory for Molecular Sciences (BNLMS) CAS Key Laboratory of Molecular Nanostructure and Nanotechnology Institute of Chemistry Chinese Academy of Sciences Beijing 100190 China

**Keywords:** charge accumulation, electron spin state, nitrogen reduction reaction, single atom catalysts

## Abstract

Understanding the relationship between the electronic state of active sites and N_2_ reduction reaction (NRR) performance is essential to explore efficient electrocatalysts. Herein, atomically dispersed Fe and Mo sites are designed and achieved in the form of well‐defined FeN_4_ and MoN_4_ coordination in polyphthalocyanine (PPc) organic framework to investigate the influence of the spin state of FeN_4_ on NRR behavior. The neighboring MoN_4_ can regulate the spin state of Fe center in FeN_4_ from high‐spin (*d* *_xy_* ^2^ *d_yz_* ^1^ *d_xz_* ^1^ $d_{z^{2}}$ ^1^ $d_{x^{2}-y^{2}}$ ^1^) to medium‐spin (*d_xy_* ^2^ *d_yz_* ^2^ *d_xz_* ^1^ $d_{z^{2}}$ ^1^), where the empty d orbitals and separate d electron favor the overlap of Fe 3d with the N 2p orbitals, more effectively activating N≡N triple bond. Theoretical modeling suggests that the NRR preferably takes place on FeN_4_ instead of MoN_4_, and the transition of Fe spin state significantly lowers the energy barrier of the potential determining step, which is conducive to the first hydrogenation of N_2_. As a result, FeMoPPc with medium‐spin FeN_4_ exhibits 2.0 and 9.0 times higher Faradaic efficiency and 2.0 and 17.2 times higher NH_3_ yields for NRR than FePPc with high‐spin FeN_4_ and MoPPc with MoN_4_, respectively. These new insights may open up opportunities for exploiting efficient NRR electrocatalysts by atomically regulating the spin state of metal centers.

## Graphical abstract

The atomically dispersed Mo–N moieties induce charge accumulation and spin‐state transition of single atom Fe sites, and the e_g_ orbital occupies one electron, which is more conducive to the Fe 3d orbitals overlap with the N 2p orbitals promoting the N_2_ reduction reaction performance.

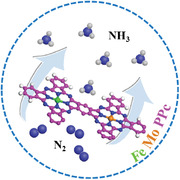

## 1 Introduction

As an important precursor to fertilizers and industrial chemicals in daily life, ammonia (NH_3_) is essential for agriculture and industry.^[^ ^1^, ^2^, ^3^, ^4^ ^]^ Nowadays, industrial synthesis of NH_3_ using N_2_ and H_2_ relies on the *Haber–Bosch* process at high temperatures (400–600 °C) and high pressure (20–40 MPa) which consumes a large amount of energy with emission of CO_2_.^[^ ^5^, ^6^, ^7^, ^8^, ^9^, ^10^, ^11^ ^]^ Among the alternative methods,^[^ ^12^, ^13^, ^14^ ^]^ the electrocatalytic N_2_ reduction reaction (NRR) using water as hydrogen source under ambient conditions has been considered as sustainable artificial N_2_ fixation to produce NH_3_.^[^ ^15^, ^16^, ^17^, ^18^, ^19^ ^]^ However, the following challenges remains for the NH_3_ production via NRR: a) the adsorption and activation of N_2_ on the catalytic sites and the difficulties in breaking strong N≡N triple bond, leading to the very low NH_3_ yield;^[^ ^20^, ^21^, ^22^ ^]^ b) the competing hydrogen evolution reaction (HER) in aqueous electrolytes, resulting in the low Faradaic efficiency (FE).^[^ ^23^, ^24^, ^25^ ^]^ Therefore, understanding the structure–activity relationship of catalytic sites for NRR is critical to the exploration of efficient electrocatalysts for feasible industrial NH_3_ production from inert N_2_ via NRR. To date, various materials, including noble‐metal‐based,^[^ ^26^ ^]^ non‐noble metal‐based,^[^ ^27^ ^]^ metal‐free,^[^ ^28^ ^]^ and single atomic materials,^[^ ^29^, ^30^ ^]^ have been investigated as electrocatalysts for NRR. Among them, single atom catalysts with the maximum of atom utilization exhibit remarkably enhanced catalytic activity and selectivity.^[^ ^31^, ^32^ ^]^

As one of the most earth‐abundant elements, Fe‐based materials have been given increasing attention as NRR catalysts.^[^ ^27^, ^33^ ^]^ According to the theoretical prediction, Mo‐based catalysts are also promising candidates for N_2_ activation.^[^ ^34^, ^35^, ^36^ ^]^ Moreover, it is notable that the long‐range coupling of hetero‐single‐atom (h‐SAs) can be used to change the electronic structure of active stie and enhance its catalytic performance. Zhao et al.^[^ ^37^ ^]^ reported that the atomically dispersed FeN_4_ and NiN_4_ coanchored on a microsized N‐doped graphitic carbon exhibited the enhanced performance for oxygen reduction reaction (ORR) through the synergetic effect. It implies that harnessing the synergy from h‐SAs would be effective for the design of NRR catalysts although the underlying mechanism for such enhancement still remains unclear.

Recent studies have reported that the electronic structure of catalytic sites can be tuned by changing the spin state of transition metals, offering the possibility for enhancing the catalytic performance. Due to the unique coordination environment, Fe possesses multiple spin states, i.e., low‐spin, medium‐spin, and high‐spin states. The Fe in the low‐spin state (no electron filling in the e_g_ orbital) has no singlet electron to pair with the intermediate.^[^ ^38^ ^]^ The Fe in the high‐spin state (e_g_ filled with two single electrons) usually displays excessive orbital overlap with the intermediates that limits the desorption of reaction product,^[^ ^39^ ^]^ resulting in inferior catalytic performance. In contrast, the Fe in the medium‐spin state (e_g_ filled with a singlet electron) can readily interact with reaction intermediates via *σ*‐bonding.^[^ ^40^ ^]^

Zhao and co‐workers^[^ ^39^ ^]^ suggested that the coexistence of Fe 3d itinerant charge and medium‐spin polarization induced by the spin‐state change of Fe in the Fe–NFe–Ni atomic pair contributed to its excellent bifunctional electrocatalytic activity for both ORR and OER. We recently found that the Mn–N could effectively activate Fe^III^ center for ORR by changing its spin state in the dual‐metal atomically dispersed Fe,Mn/N–C catalyst.^[^ ^38^ ^]^ However, the electronic configurations of ground‐state O_2_ and N_2_ molecules are completely different according to the molecular orbital theory. O_2_ has a spin‐triplet state with two unpaired electrons, while N_2_ is diamagnetic with all electrons paired up.^[^ ^28^ ^]^ Thus, the spin regulation and its influence on NRR performance would be significantly different from ORR/OER, which have been not investigated and revealed yet although it might be an efficient strategy to accelerate the NRR kinetics.

To fill puzzle, we herein designed and successfully fabricated a well‐defined Fe–Mo h‐SA NRR electrocatalyst with atomically dispersed FeN_4_ and MoN_4_ coordination sites coanchored in polyphthalocyanine organic framework (FeMoPPc) by a low‐temperature melt polymerization. Density functional theory (DFT) calculation and experimental results reveal that the introduction of Mo SAs induced the charge accumulation on the Fe active sites, which promotes the adsorption and activation of N_2_. Importantly, the change of Fe spin state from high to medium spin simultaneously facilitated the potential determining step (PDS) of NRR, the first protonation step. Due to the synergistic effect of charge accumulation and spin redistribution, the difficulties in activating N≡N triple bond and protonation hydrogenation were effectively mitigated. As a result, the obtained FeMoPPc with medium‐spin Fe center exhibited 2.0 and 9.0 times higher Faradic efficiencies and 2.0 and 17.2 times higher NH_3_ yields for NRR than FePPc with high‐spin Fe and MoPPc with Mo active center, respectively.

## 2 Results and Discussion

**Figure 1:**
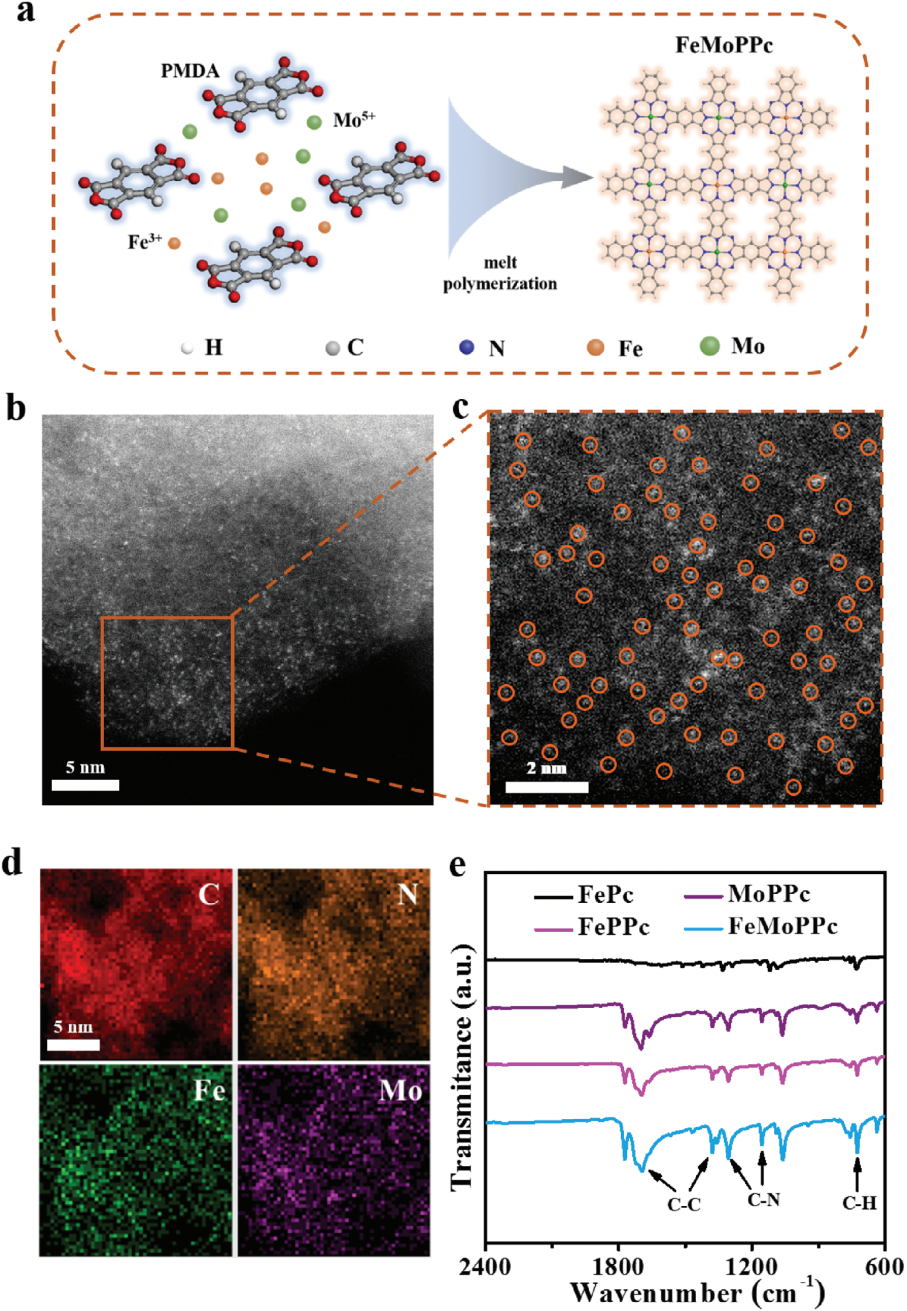
Synthetic illustration, TEM, and IR characterizations of FeMoPPc catalyst. a) Synthetic procedure of FeMoPPc. b) HAADF‐STEM image of FeMoPPc. c) Enlarged view of selected area HAADF‐STEM image of FeMoPPc. d) EDS elemental mapping of C, N, Fe, and Mo in FeMoPPc. e) FTIR spectra of FePc, FePPc, MoPPc, and FeMoPPc.

As illustrated in **Figure** **1**a, FeMoPPc was synthesized by a low‐temperature solvent‐free solid‐phase polymerization of pyromellitic dianhydride, urea, FeCl_3_, and MoCl_3_ with the (NH_4_)_2_Mo_2_O_7_ as initiator (see the details in the Supporting Information). Typically, pyromellitic dianhydride and urea were employed as organic phthalocyanine precursor and metal chlorides were used as metal source, respectively. The above chemicals were first mixed and ground uniformly in an agate mortar, and then heated at 220 °C for 3 h for polymerization process. Specifically, the anhydrides group in the pyromellitic dianhydride reacts with the amino group of urea to form the imide group, which continues to react with the amino group of urea and give the iminoisoindoline. Meanwhile, Fe ions and Mo ions were coordinated with the amino groups of iminoisoindoline monomer and subsequently form phthalocyanine network polymer containing Fe(Mo)N_4_.^[^ ^41^, ^42^, ^43^ ^]^ The proper diameter of the ring cavity in the polyphthalocyanine structure can accommodate Fe and Mo metal atoms, forming a metal phthalocyanine coordination polymer with homogeneous distributions of Fe and Mo. The control samples FePPc and MoPPc were prepared in parallel excepting for using Fe or Mo source alone instead of both for FeMoPPc.

Field‐emission scanning electron microscopy (SEM) and transmission electron microscopy (TEM) images of FeMoPPc (Figure S1a,b, Supporting Information) show a fluffy porous morphology,^[^ ^41^ ^]^ similar to that of KB (Figure S2, Supporting Information). No metal‐based particles or aggregations were observed in the TEM image (Figure S1c, Supporting Information), indicating the uniform loading of FeMoPPc on the substrate. The high‐resolution TEM (HR‐TEM) image (Figure S1d, Supporting Information) and the corresponding selected area electron diffraction (SAED) image (inset in Figure S1d in the Supporting Information) demonstrate the amorphous structure for FeMoPPc. Field‐emission scanning electron microscopy energy dispersive spectroscopic (SEM‐EDS) elemental mapping displays C, N, Fe, and Mo are uniformly distributed, indicating that FeMoPPc is evenly loaded on carbon substrate (Figure S3, Supporting Information). This result is consistent with X‐ray diffraction (XRD) measurements (Figure S4, Supporting Information), indicating the absence of the long‐range order in FeMoPPc. Some individual bright points can be observed in the high‐angle annular dark‐field scanning TEM (HAADF‐STEM) images (Figure 1b,c), suggesting that Fe and Mo should exist in the form of single atoms in FeMoPPc. EDS elemental mapping displays the homogeneous distribution of C, N, Fe, and Mo elements (Figure 1d). The FT‐IR spectra (Figure 1e) of FePPc, FeMoPPc, and MoPPc show obvious phthalocyanine skeletal vibration in the range of 700–1600 cm^−1^,^[^ ^8^ ^]^ and these results suggest the successful formation of metal polyphthalocyanine frameworks. The Brunauer–Emmett–Teller (BET) specific surface area of FeMoPPc on KB is much lower than that of KB itself (63.3 cm^2^ g^−1^ vs 1347.2 cm^2^ g^−1^) (Figure S5, Supporting Information), further evidencing the successful loading of FeMoPPc on KB.

The chemical states and coordination environment of Fe and Mo atoms in FeMoPPc were further determined by X‐ray photoelectron spectroscopy (XPS) and X‐ray absorption spectroscopy (XAS). XPS spectrum of FeMoPPc confirms the presence of Fe, Mo, C, and N elements (Figure S6a, Supporting Information). The high‐resolution Fe 2p XPS exhibits two peaks at 709.7 and 722.2 eV (Figure S6b, Supporting Information), corresponding to Fe 2p_3/2_ and Fe 2p_1/2_, respectively. The estimated valence state of Fe is +2.^[^ ^44^ ^]^ There are two shakeup satellites peaks (denoted as “Sat.”) centered at 709.7 and 722.2 eV. In Mo 3d XPS (Figure S6c, Supporting Information), the split peaks centered at 230.4 and 233.5 eV can be assigned to the Mo 3d_5/2_ and Mo 3d_3/2_ of Mo^4+^,^[^ ^45^ ^]^ respectively. The N 1s XPS (Figure S6d, Supporting Information) reveals the coexistence of five types of N species for FeMoPPc, including oxidized N (402.6 eV), pyrrolic N (400.8 eV), Fe–N (399.8 eV), pyridinic N (398.5 eV), and Mo–N (397.4 eV).^[^ ^46^ ^]^ The content of Fe and Mo detected by inductively coupled plasma atomic emission spectroscopy (ICP‐AES) analysis are 1.28 and 5.83 wt%, respectively (see Table S1 in the Supporting Information for more details).

**Figure 2:**
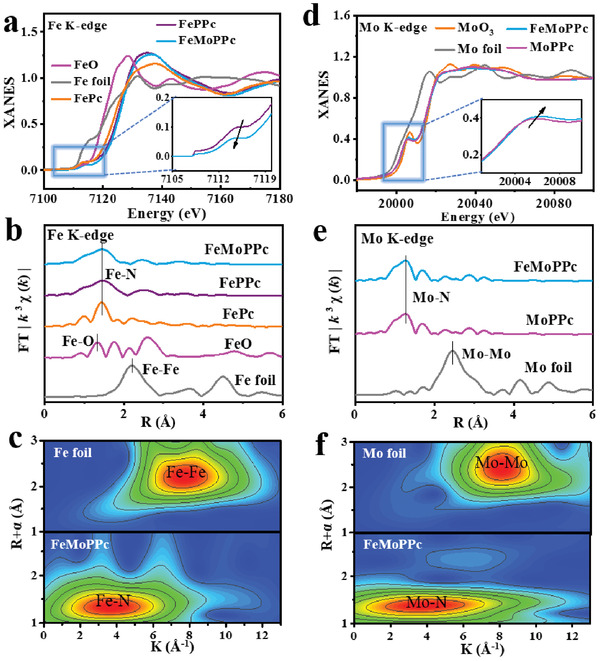
XAS spectrum of the catalysts. a) Fe K‐edge XANES and b) Fourier‐transformed for the catalyst FeMoPPc and other standard samples. c) Wavelet transform of Fourier‐transformed EXAFS data of Fe foil, FeMoPPc. d) Mo K‐edge XANES and e) Fourier‐transformed for the catalyst FeMoPPc and other standard samples. *R* is the distance in Ångströms (1 Å = 10^−10^ m); *k* is the wave number. f) Wavelet transform of Fourier‐transformed EXAFS data of Mo foil, FeMoPPc.

The normalized Fe K‐edge X‐ray absorption near edge structure (XANES) spectra display that the Fe states of FeMoPPc and FePPc are similar to that of FePc (**Figure** **2**a).^[^ ^47^, ^48^ ^]^ It is worth noting that the absorption edge of FeMoPPc shifts slightly to the lower energy compared to that of FePPc (inset in Figure 2a), indicating that the introduction of Mo has an effect on the coordination environment of Fe. In reference to FeO and Fe foil, the Fourier‐transformed extended X‐ray absorption fine structure (FT‐EXAFS) spectra of the FeMoPPc shows a main peak at 1.4 Å belonging to the Fe–N scattering paths while no Fe‐Fe interaction peak located at >2.2 Å and Fe‐O peak at 1.2 Å were observed (Figure 2b), confirming the atomic dispersion of Fe in FeMoPPc. Wavelet transforms of Fe K‐edge EXAFS oscillations were performed to further investigate the status of Fe atoms. WT analysis of FeMoPPc spectrum shows only one intensity maximum at about 4.0 Å^−1^ (Figure 2c), very similar to the reference FePc (at ≈4.0 Å^−1^) (Figure S7a, Supporting Information) but distinct from the Fe foil (at ≈7.5 Å^−1^). These results suggest that FeMoPPc has the FeN_4_ structure like that in FePc. Using this structural model, all the fitted Fe K‐edge EXAFS in R space (blue line) and k space (blue line) match well with the experimental data (red circles) (Figure S7b,c, Supporting Information). The fitting results show that the isolated Fe atom coordinates with N atoms in an average coordination number of 4 and an average Fe—N bond length is 1.96 Å (see Table S2 in the Supporting Information for more details). Meanwhile, two O atoms are adsorbed on the Fe atom in side‐on mode, which is well compatible with WT.

Moreover, the Mo K‐edge XANES spectra display that FeMoPPc and MoPPc have similar Mo edge feature (Figure 2d), indicating the chemical environment of Mo–N coordination.^[^ ^29^ ^]^ In the FT‐EXAFS spectra (Figure 2e), FeMoPPc shows a main peak belonging to the Mo–N scattering paths located at 1.3 Å while no Mo–Mo peak is detected at about 2.5 Å,^[^ ^29^ ^]^ confirming the atomic dispersion of Mo. WT analyses of Mo K‐edge EXAFS oscillations corroborate that Mo exists in the form of single atomic Mo–N in FeMoPPc in view of one intensity maximum at about 4.0 Å^−1^ (Figure 2f; Figure S8a, Supporting Information) which is distinct from the feature of Mo foil (≈8.0 Å^−1^). All the fitted Mo K‐edge EXAFS in R space (blue line) and k space fitting curve (blue line) support the proposed atomic Mo–N coordination structure (Figure S8b,c, Supporting Information). The average coordination number is around 4 and an average Mo—N bond length is ≈1.76 Å (Table S2, Supporting Information).

**Figure 3:**
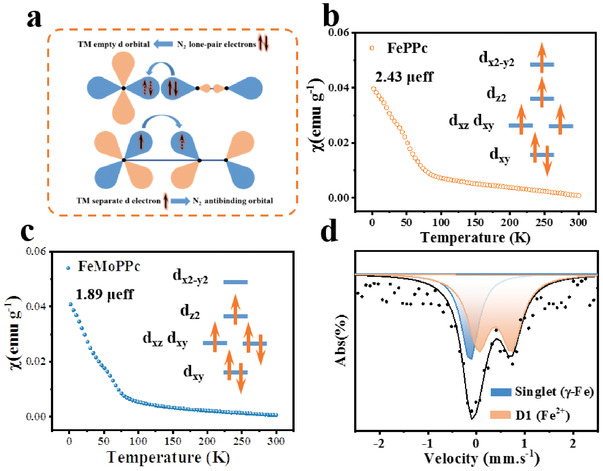
Magnetic susceptibility and ^57^Fe Mössbauer spectroscopy of the catalysts. a) Simplified schematic of N_2_ bonding to transition metals. b,c) Magnetic susceptibility of b) FePPc and c) FeMoPPc. d) Room‐temperature ^57^Fe Mössbauer spectrum of FeMoPPc. The data (scattered dots) are shown with the best fit (black dash line) and the deconvolutions of two components as indicated by the legend.

Theoretically, the NRR performance on a transition metal (TM)‐based catalyst is related to the occupancy rate of its d orbitals.^[^ ^49^ ^]^ On the one hand, metal center should have empty d orbitals to accept the lone‐pair electrons of N_2_. On the other hand, the separate d electron is expected to contribute to the antibonding orbital of N_2_, weakening the strong N≡N triple bond (*π*‐backdonation) (**Figure** **3**a).^[^ ^50^ ^]^ In this regard, the zero‐field cooling (ZFC) temperature‐dependent magnetic susceptibility measurements were carried out to reveal the electronic configuration of the d orbitals of Fe in FeMoPPc. Figure 3b,c shows a nearly temperature‐independent paramagnetism for both FePPc and FeMoPPc. Notably, the calculated effective magnetic moment^[^ ^38^ ^]^ of Fe sites for adsorption goes from 2.43 to 1.89 μeff after the introduction of Mo. Moreover, the density of states (DOS) of FeMoPPc undergoes partial spin splitting compared with FePPc, indicating that the spin state of Fe species in FeMoPPc is reduced (Figure S9, Supporting Information).^[^ ^51^ ^]^ It suggests that Fe center changes from high spin (*d_xy_* ^2^ *d_yz_* ^1^ *d_xz_* ^1^ $d_{z^{2}}$ ^1^ $d_{x^{2}-y^{2}}$ ^1^) to medium spin (*d_xy_* ^2^ *d_yz_* ^2^ *d_xz_* ^1^ $d_{z^{2}}$ ^1^) after adding Mo, where it has both empty d orbitals and separate *d* electron.^[^ ^52^, ^53^ ^]^ Such Fe 3d orbitals would effectively overlap with the N 2p orbital while weakening the N≡N triple bond.

^57^Fe Mössbauer spectroscopy of FeMoPPc was further recorded to analyze the Fe state. As shown in Figure 3d, a singlet and a doublet (D1) can be fitted. The singlet state is usually assigned to *γ*‐Fe. With the largest quadrupole splitting (QS) value, D1 can be assigned to FePc‐like medium‐spin Fe^II^N_4_ species.^[^ ^48^ ^]^ The quantitative analysis shows that the content of D1 is 66.3% (Table S3, Supporting Information), indicating that Fe^II^ mainly exists in medium spin in FeMoPPc. Otherwise, if Fe is in high spin where two unpaired electrons sit in e_g_ orbital, Fe 3d and N 2p orbital will excessively overlap, resulting in too strong interaction and difficulty in the first hydrogenation step. Based the above data, the neighboring MoN_4_ sites appreciably regulates the spin state of Fe center from high spin to medium spin, promoting *π*‐backdonation process and the first hydrogenation of N_2_, as discussed later.

**Figure 4:**
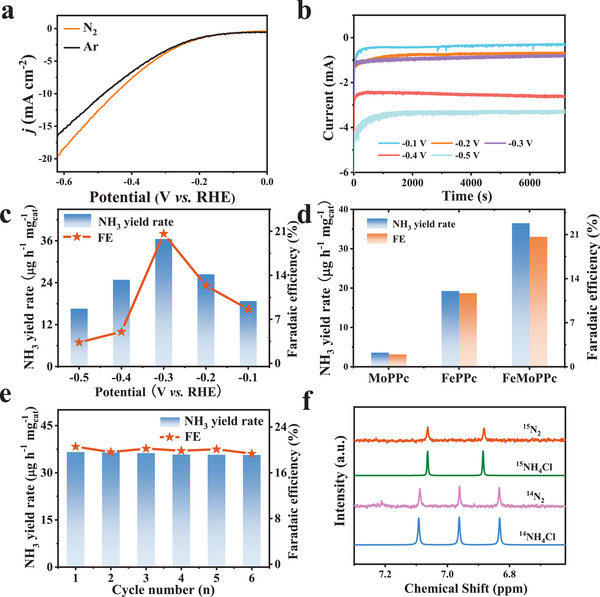
NRR electrochemical performances of FeMoPPc in 0.1 m KOH. a) Linear sweep voltammetry curves in N_2_ (orange line) and Ar‐saturated (black line) KOH solutions. b) Chronoamperometry results at different potentials. c) NH_3_ yield rate and Faradaic efficiency at the corresponding potentials. d) NH_3_ yield rate and Faradaic efficiency of MoPPc, FePPc, and FeMoPPc in N_2_‐saturated 0.1 m KOH solution at −0.3 V for 2h, respectively. e) Cycling test of FeMoPPc for the NRR at −0.3 V. f) ^1^H NMR analysis of the electrolyte fed by ^14^N_2_ and ^15^N_2_ after the electrolytic reaction.

Inspiring by this change in spin state and motivation of revealing its effects on the electrocatalytic NRR performance, we systematically evaluated the catalytic performances of FeMoPPc, FePPc, and MoPPc for NRR in an H‐type cell (Figure S10, Supporting Information) using N_2_ or Ar‐saturated 0.1 m KOH solution as the electrolyte.^[^ ^54^ ^]^ All the potentials hereinafter were referred to the reversible hydrogen electrode (RHE). As shown in **Figure** **4**a, an obvious shift in the linear sweep voltammetry (LSV) curves was detected for FeMoPPc when switching the electrolyte from Ar‐saturated solution to N_2_‐saturated one, implying its possible activity for NRR. Figure S11 in the Supporting Information shows the LSV curves of the FePPc and MoPPc and N_2_‐saturated 0.1 m KOH solution. The yields of NH_3_ were evaluated by the indophenol blue method using NH_4_Cl solutions as standards.^[^ ^26^ ^]^ As shown in Figure S12 (Supporting Information), under alkaline conditions, the absorbance of standard solutions in various concentrations can be recorded at a wavelength of 655 nm. The fitting curves give excellent linear correlations of absorbances with NH_4_Cl concentrations under both alkaline and acidic electrolytes (Figure S13, Supporting Information), enabling the reliable determination of NH_3_ yields for NRR experiments.

During the synthetic process, the atomic ratio of Fe to Mo in FeMoPPc was adjusted by simply changing the amounts of source FeCl_3_ and MoCl_5_. XPS was carried out to analyze the atomic ratios of Fe to Mo in the samples (Table S4, Supporting Information). For example, 0.5:1 of FeCl_3_:MoCl_5_ gives ≈0.71:1 of Fe/Mo in FeMoPPc. Electrochemical NRR tests were then performed on a series of FeMoPPc samples with different Fe:Mo ratios. It was found that when using 0.5:1 of FeCl_3_:MoCl_5_ the NH_3_ yield and FE on FeMoPPc are the highest (Figure S14, Supporting Information). The chronoamperometric curves were collected over 2h under various testing potentials ranging from −0.1 to −0.5 V, and the NRR measurements demonstrate that NH_3_ yield and FE on FeMoPPc reach the highest values of 36.33 µg h^−1^ mg^−1^_cat_ and 20.62% at −0.3 V, respectively (Figure 4b,c; Figure S15, Supporting Information). Compared with the previous reports, such performance ranks top tier among Fe‐based or Mo‐based catalysts (Figure S16 and Table S5, Supporting Information). More impressively, these values are 2.0 times higher than those on FePPc where the Fe center is in medium spin instead of high spin (Figure 4d), suggesting the spin state of Fe center significantly affects the NH_3_ yield and FE. Moreover, it is worth noting that NH_3_ yield and FE on FeMoPPc are 9.0 and 17.2 times higher than those on MoPPc with only MoN_4_ sites (Figure 4d), implying the FeN_4_ sites may be much more active than MoN_4_ for NRR. The cycling tests were further performed to probe the durability. As shown in Figure 4e, no obvious variation for the NH_3_ yields and FE in successive six cycling is observed. Furthermore, the FeMoPPc working electrode outputs stable current densities of NRR in this cycling tests (Figure S17, Supporting Information). All these results corroborate the superior durability of FeMoPPc for NRR.

Subsequently, the method of Watt and Chrisp^[^ ^26^ ^]^ was used to probe the possible by‐product hydrazine (N_2_H_4_) in the electrolyte. As shown in Figure S18 (Supporting Information), a series of standard N_2_H_4_ solutions with different concentrations were used to plot the calibration curves of N_2_H_4_ in KOH solutions and HCl solutions (Figure S19, Supporting Information), respectively. The testing results prove that no N_2_H_4_ was produced in the system (Figure S20, Supporting Information), indicating that FeMoPPc has excellent selectivity to NH_3_ production via NRR.

To reveal if the detected NH_3_ is truly produced by electrocatalytic conversion of N_2_ flowed into the electrolytic cell rather than from possible contaminations (such as gas source, human breath, electrolyte, and environment, etc.), a series of control experiments were carried out. As shown in Figures S21 and S22 (Supporting Information), negligible NH_3_ were detected for the control experiments with a bare carbon paper with the N_2_‐saturated electrolyte (carbon paper‐N_2_), FeMoPPc with the Ar‐saturated electrolyte (FeMoPPc‐Ar), open circuit or a KOH background at −0.3 V. This suggests that the NH_3_ was generated from electroreduction of supplied N_2_ by FeMoPPc catalyst. To give an unambiguous conclusion, isotopic labeling experiments were further performed and confirmed that the N in NH_3_ product originated from the supplied N_2_. As indicated by the ^1^H nuclear magnetic resonance (NMR) spectra (Figure 4f), triplet coupling for ^14^NH_4_^+^ and doublet coupling for ^15^NH_4_^+^ were clearly detected when ^14^N_2_ and ^15^N_2_ were bubbled over the cathode, respectively. These signals are in well consistence with those recorded with standard solutions of ^14^NH_4_Cl and ^15^NH_4_Cl. These results reliably suggest that the detected NH_3_ is truly from NRR on FeMoPPc.

**Figure 5:**
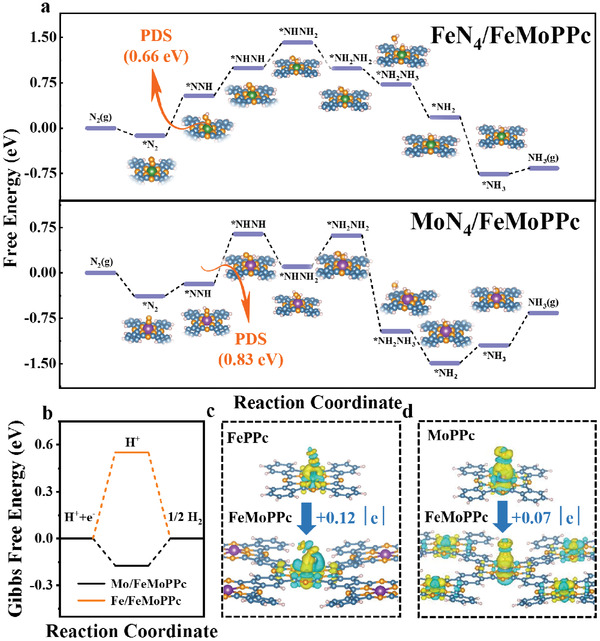
DFT calculations of the NRR activity on FeMoPPc catalysts. a) Different free‐energy diagrams for the NRR on Fe atoms of FeMoPPc and Mo atoms of FeMoPPc. b) Different free‐energy diagrams for the HER on Fe atoms of FeMoPPc and Mo atoms of FeMoPPc. c) The charge density differences calculations of N_2_ bonded to Fe atoms of FePPc and FeMoPPc (the electron excess area and electron deficiency area are represented by yellow and blue‐green, respectively). d) The charge density differences calculations of the N_2_ bonded to Mo atoms of MoPPc and FeMoPPc.

DFT calculations were subsequently performed to unveil the underlying mechanism of the overall NRR on such bimetallic Fe–Mo h‐SAs coanchored in polyphthalocyanine organic framework. Specific DFT calculation information and constant settings (Table S6, Supporting Information) can be seen in Supporting Information. According to experimental results, FeN_4_ and MoN_4_ sites were proposed as the structural models (Figure S23, Supporting Information). **Figure** **5**a shows the calculated Gibbs free energy diagrams for NRR on FeN_4_ and MoN_4_ sites in FeMoPPc via the alternating pathways (Figure S24, Supporting Information). It is seen that the first hydrogenation process (*N_2_→*NNH) appears to be the PDS for FeN_4_ site with an energy barrier of 0.66 eV. In contrast, The PDS on MoN_4_ site is the second hydrogenation process (*NNH→*NHNH_2_) with an energy barrier of 0.83 eV (Figure 5a; Figure S25, Supporting Information). Moreover, the desorption of NH_3_ on FeN_4_ site only needs to overcome a small energy barrier of 0.09 eV while that on MoN_4_ site shows a much higher energy barrier (0.54 eV). These results suggest that FeN_4_ site is more active than MoN_4_ site in FeMoPPc for NRR, rationalizing the fact that FeMoPPc exhibits 9.0 times higher FE and 17.2 times higher NH_3_ yields for NRR than MoPPc with MoN_4_ sites only. As shown in Figure S26 (Supporting Information), the DFT calculation of FeN_4_ site in FePPc has been performed. The PDS on FeN_4_ site is 1.42 eV, which is larger than FeN_4_ site of FeMoPPc, indicating that the addition of Mo promotes the NRR process on FeN_4_ site. In view of the Fe spin‐state analysis, it might be ascribed to the fact that the introduction of MoN_4_ moieties causes the transition of Fe spin state from high spin (*d_xy_* ^2^ *d_yz_* ^1^ *d_xz_* ^1^ $d_{z^{2}}$ ^1^ $d_{x^{2}-y^{2}}$ ^1^) to medium spin (*d_xy_* ^2^ *d_yz_* ^2^ *d_xz_* ^1^ $d_{z^{2}}$ ^1^). Moreover, the projected density of states (PDOS) results shows that the d‐band center of Fe/FeMoPPc occurs positive shift compared to Fe/FePPc,^[^ ^55^ ^]^ indicating that it is easier to adsorb N_2_ and coincide with N p orbital (Figure S27, Supporting Information).

HER is known to be the main competitive reaction against NRR. Therefore, the HER activity of the two active sites was also investigated. For dual component active sites of FeMoPPc, one with △*G*_H_<0 (△*G*_H‐negative_, strong H adsorption) and another one with △*G*_H_>0 (△*G*_H‐positive_, weak H adsorption), hydrogen spillover phenomenon occurs from the surface with △*G*_H‐negative_ to the surface with △*G*_H‐positive_.^[^ ^56^ ^]^ As shown in Figure 5b, Gibbs free energy diagrams display that the first protonation process of HER occurred spontaneously on Mo‐SAs, while for Fe‐SAs, it is nonspontaneous, indicating that the ability of H atoms to bind to Mo is stronger than Fe.^[^ ^57^ ^]^ Therefore, the H intermediate on the Mo sites migrates to the Fe sites through spillover, assists the N_2_ hydrogenation on the Fe sites, and synergistically enhances the NRR activity. As shown in Figure 5c,d and Figure S28 (Supporting Information), the charge density difference calculations show that more electrons can be donated to the *N_2_ species from Fe sites than from Mo sites. The Fe atoms in the FeMoPPc system have an increase of +0.12 |e| electrons compared to the FePPc system, while +0.07 |e| electrons are added for the Mo atoms in the FeMoPPc system compared to the MoPPc system. When the number of electrons on the metal site increases, the number of charges flowing to the N≡N bond to form the M—N bond increases, which is more conducive to the adsorption and activation of N_2_.^[^ ^58^ ^]^ It indicates that the adsorption and activation of N_2_ by Fe sites are enhanced after adding metal Mo.

## 3 Conclusion

In summary, a h‐SA NRR electrocatalyst with FeN_4_ and MoN_4_ coordination coanchored to polyphthalocyanine organic framework (POF) by low‐temperature melt polymerization, which demonstrated that the Fe‐SAs and Mo‐SAs active sites synergistically promote the NRR process. uncover that the spin‐state formation of 3d orbitals in transition metal ions. Importantly, both experiments and DFT calculations revealed that the addition of Mo‐SAs can induce electrons to accumulate on Fe, which was more conducive to the adsorption of N_2_. As a result, Fe changed from high spin to medium spin with a decrease of unpaired electrons, which better coincided with *N_2_ unpaired electrons, thereby promoting the first hydrogenation process. Thereby, FeMoPPc exhibits 2.0 and 9.0 times higher Faradaic efficiency and 2.0 and 17.2 times higher NH_3_ yields for NRR than FePPc and MoPPc, respectively. This work potentially provides a rational design principle for optimizing noble‐metal‐free NRR electrocatalysts by smartly altering the specific local environment.

## Conflict of Interest

The authors declare no conflict of interest.

## Author Contributions

Z.J.N. conceived the project and idea. W.Y.J. performed the materials preparation, characterization, performance testing, and wrote the article. H.Y.F. performed NEXFAS experiments and data fitting with the discussion with Z.J.N. C.W.Z., Y.G.G., L.J.L., X.H.C., G.K., L.M.L., Z.S.Y., Q.G., L.B.A., and H.J.S. assisted in the materials preparation and data analysis. Y.P.F. performed the theoretical calculations. Z.J.N. supervised the project.

## Supporting information

Supporting Information

## Data Availability

Research data are not shared.

## References

[1] R. F. Service , Science 2014, 345, 610. 25104365 10.1126/science.345.6197.610

[2] L. Li , C. Tang , X. Cui , Y. Zheng , X. Wang , H. Xu , S. Zhang , T. Shao , K. Davey , S. Qiao , Angew. Chem., Int. Ed. 2021, 60, 14131. 10.1002/anie.202104394 33855782

[3] S. Liu , T. Qian , M. Wang , H. Ji , X. Shen , C. Wang , C. Yan , Nat. Catal. 2021, 4, 322.

[4] T. Wu , H. Zhao , X. Zhu , Z. Xing , Q. Liu , T. Liu , S. Gao , S. Lu , G. Chen , A. M. Asiri , Y. Zhang , X. Sun , Adv. Mater. 2020, 32, 2000299. 10.1002/adma.202000299 32567074

[5] R. Schlögl , Angew. Chem., Int. Ed. 2003, 115, 2050.

[6] Z. Han , C. Choi , S. Hong , T.‐S. Wu , Y.‐L. Soo , Y. Jung , J. Qiu , Z. Sun , Appl. Catal., B 2019, 257, 117896.

[7] J. Guo , C. Y. Lin , Z. Xia , Z. Xiang , Angew. Chem., Int. Ed. 2018, 57, 12567. 10.1002/anie.201808226 30051963

[8] N. Han , Y. Wang , L. Ma , J. Wen , J. Li , H. Zheng , K. Nie , X. Wang , F. Zhao , Y. Li , J. Fan , J. Zhong , T. Wu , D. J. Miller , J. Lu , S.‐T. Lee , Y. Li , Chem 2017, 3, 652.

[9] W. Guo , K. Zhang , Z. Liang , R. Zou , Q. Xu , Chem. Soc. Rev. 2019, 48, 5658. 31742279 10.1039/c9cs00159j

[10] Y. Luo , G.‐F. Chen , L. Ding , X. Chen , L.‐X. Ding , H. Wang , Joule 2019, 3, 279.

[11] S. Z. Andersen , M. J. Statt , V. J. Bukas , S. G. Shapel , J. B. Pedersen , K. Krempl , M. Saccoccio , D. Chakraborty , J. Kibsgaard , P. C. K. Vesborg , J. Nørskov , I. Chorkendorff , Energy Environ. Sci. 2020, 13, 4291.

[12] J. Deng , J. A. Iñiguez , C. Liu , Joule 2018, 2, 846.

[13] K. Han , J. Luo , Y. Feng , L. Xu , W. Tang , Z. L. Wang , Energy Environ. Sci. 2020, 13, 2450.

[14] Y. Ren , C. Yu , X. Tan , H. Huang , Q. Wei , J. Qiu , Energy Environ. Sci. 2021, 14, 1176.

[15] M. Kitano , Y. Inoue , Y. Yamazaki , F. Hayashi , S. Kanbara , S. Matsuishi , T. Yokoyama , S. W. Kim , M. Hara , H. Hosono , Nat. Chem. 2012, 4, 934. 23089869 10.1038/nchem.1476

[16] V. Kyriakou , I. Garagounis , E. Vasileiou , A. Vourros , M. Stoukides , Catal. Today 2017, 286, 2.

[17] C. Tang , S. Z. Qiao , Chem. Soc. Rev. 2019, 48, 3166. 31107485 10.1039/c9cs00280d

[18] S. Z. Andersen , V. Colic , S. Yang , J. A. Schwalbe , A. C. Nielander , J. M. McEnaney , K. Enemark‐Rasmussen , J. G. Baker , A. R. Singh , B. A. Rohr , M. J. Statt , S. J. Blair , S. Mezzavilla , J. Kibsgaard , P. C. K. Vesborg , M. Cargnello , S. F. Bent , T. F. Jaramillo , I. E. L. Stephens , J. K. Norskov , I. Chorkendorff , Nature 2019, 570, 504. 31117118 10.1038/s41586-019-1260-x

[19] B. Yang , W. Ding , H. Zhang , S. Zhang , Energy Environ. Sci. 2021, 14, 672.

[20] N. Cao , G. Zheng , Nano Res. 2018, 11, 2992.

[21] H. K. Chae , D. Y. Siberio‐Perez , J. Kim , Y. Go , M. Eddaoudi , A. J. Matzger , M. O'Keeffe , O. M. Yaghi , Nature 2004, 427, 523. 14765190 10.1038/nature02311

[22] Y.‐C. Hao , Y. Guo , L.‐W. Chen , M. Shu , X.‐Y. Wang , T.‐A. Bu , W.‐Y. Gao , N. Zhang , X. Su , X. Feng , J.‐W. Zhou , B. Wang , C.‐W. Hu , A.‐X. Yin , R. Si , Y.‐W. Zhang , C.‐H. Yan , Nat. Catal. 2019, 2, 448.

[23] M. Falcone , L. Barluzzi , J. Andrez , F. Fadaei Tirani , I. Zivkovic , A. Fabrizio , C. Corminboeuf , K. Severin , M. Mazzanti , Nat. Chem. 2019, 11, 154. 30420774 10.1038/s41557-018-0167-8

[24] Y. X. Lin , S. N. Zhang , Z. H. Xue , J. J. Zhang , H. Su , T. J. Zhao , G. Y. Zhai , X. H. Li , M. Antonietti , J. S. Chen , Nat. Commun. 2019, 10, 4380. 31558716 10.1038/s41467-019-12312-4 PMC6763479

[25] Y. Ma , T. Yang , H. Zou , W. Zang , Z. Kou , L. Mao , Y. Feng , L. Shen , S. J. Pennycook , L. Duan , X. Li , J. Wang , Adv. Mater. 2020, 32, 2002177. 10.1002/adma.202002177 32627888

[26] Z. H. Xue , S. N. Zhang , Y. X. Lin , H. Su , G. Y. Zhai , J. T. Han , Q. Y. Yu , X. H. Li , M. Antonietti , J. S. Chen , J. Am. Chem. Soc. 2019, 141, 14976. 31523954 10.1021/jacs.9b07963

[27] T. Wu , X. Zhu , Z. Xing , S. Mou , C. Li , Y. Qiao , Q. Liu , Y. Luo , X. Shi , Y. Zhang , X. Sun , Angew. Chem., Int. Ed. 2019, 58, 18449. 10.1002/anie.201911153 31549471

[28] Y. Yang , L. Zhang , Z. Hu , Y. Zheng , C. Tang , P. Chen , R. Wang , K. Qiu , J. Mao , T. Ling , S. Z. Qiao , Angew. Chem., Int. Ed. 2020, 59, 4525. 10.1002/anie.201915001 31950550

[29] L. Han , X. Liu , J. Chen , R. Lin , H. Liu , F. Lu , S. Bak , Z. Liang , S. Zhao , E. Stavitski , J. Luo , R. R. Adzic , H. L. Xin , Angew. Chem., Int. Ed. 2019, 58, 2321. 10.1002/anie.201811728 30548557

[30] J. Yao , J. Yan , Chem 2020, 6, 808.

[31] S. Ji , Y. Chen , X. Wang , Z. Zhang , D. Wang , Y. Li , Chem. Rev. 2020, 120, 11900. 32242408 10.1021/acs.chemrev.9b00818

[32] H. Tao , C. Choi , L.‐X. Ding , Z. Jiang , Z. Han , M. Jia , Q. Fan , Y. Gao , H. Wang , A. W. Robertson , S. Hong , Y. Jung , S. Liu , Z. Sun , Chem 2019, 5, 204.

[33] L. Hu , A. Khaniya , J. Wang , G. Chen , W. E. Kaden , X. Feng , ACS Catal. 2018, 8, 9312.

[34] G. Qing , R. Ghazfar , S. T. Jackowski , F. Habibzadeh , M. M. Ashtiani , C. P. Chen , M. R. Smith II , T. W. Hamann , Chem. Rev. 2020, 120, 5437. 32459470 10.1021/acs.chemrev.9b00659

[35] H. Su , L. Chen , Y. Chen , R. Si , Y. Wu , X. Wu , Z. Geng , W. Zhang , J. Zeng , Angew. Chem., Int. Ed. 2020, 59, 20411. 10.1002/anie.202009217 32743842

[36] J. Li , S. Chen , F. Quan , G. Zhan , F. Jia , Z. Ai , L. Zhang , Chem 2020, 6, 885.

[37] Z. Zhu , H. Yin , Y. Wang , C. H. Chuang , L. Xing , M. Dong , Y. R. Lu , G. Casillas‐Garcia , Y. Zheng , S. Chen , Y. Dou , P. Liu , Q. Cheng , H. Zhao , Adv. Mater. 2020, 32, 2004670. 10.1002/adma.202004670 32939887

[38] G. Yang , J. Zhu , P. Yuan , Y. Hu , G. Qu , B. A. Lu , X. Xue , H. Yin , W. Cheng , J. Cheng , W. Xu , J. Li , J. Hu , S. Mu , J. N. Zhang , Nat. Commun. 2021, 12, 1734. 33741940 10.1038/s41467-021-21919-5 PMC7979714

[39] H. Li , J. Wang , R. Qi , Y. Hu , J. Zhang , H. Zhao , J. Zhang , Y. Zhao , Appl. Catal., B 2021, 285, 119778.

[40] Z. Li , Z. Zhuang , F. Lv , H. Zhu , L. Zhou , M. Luo , J. Zhu , Z. Lang , S. Feng , W. Chen , L. Mai , S. Guo , Adv. Mater. 2018, 30, 1803220. 10.1002/adma.201803220 30260517

[41] Y. Pan , S. Liu , K. Sun , X. Chen , B. Wang , K. Wu , X. Cao , W. C. Cheong , R. Shen , A. Han , Z. Chen , L. Zheng , J. Luo , Y. Lin , Y. Liu , D. Wang , Q. Peng , Q. Zhang , C. Chen , Y. Li , Angew. Chem., Int. Ed. 2018, 57, 8614. 10.1002/anie.201804349 29749097

[42] J. Chen , K. Zou , P. Ding , J. Deng , C. Zha , Y. Hu , X. Zhao , J. Wu , J. Fan , Y. Li , Adv. Mater. 2019, 31, 1805484. 10.1002/adma.201805484 30393896

[43] S. Yang , Y. Yu , M. Dou , Z. Zhang , F. Wang , J. Am. Chem. Soc. 2020, 142, 17524. 32942851 10.1021/jacs.0c07249

[44] H. Cheng , P. Cui , F. Wang , L. X. Ding , H. Wang , Angew. Chem., Int. Ed. 2019, 58, 15541. 10.1002/anie.201910658 31502747

[45] Y. Guo , Z. Yao , B. J. J. Timmer , X. Sheng , L. Fan , Y. Li , F. Zhang , L. Sun , Nano Energy 2019, 62, 282.

[46] G. Chen , P. Liu , Z. Liao , F. Sun , Y. He , H. Zhong , T. Zhang , E. Zschech , M. Chen , G. Wu , J. Zhang , X. Feng , Adv. Mater. 2020, 32, 1907399. 10.1002/adma.201907399 31944436

[47] Y. Chen , S. Ji , S. Zhao , W. Chen , J. Dong , W. C. Cheong , R. Shen , X. Wen , L. Zheng , A. I. Rykov , S. Cai , H. Tang , Z. Zhuang , C. Chen , Q. Peng , D. Wang , Y. Li , Nat. Commun. 2018, 9, 5422. 30575726 10.1038/s41467-018-07850-2 PMC6303331

[48] W. Liu , L. Zhang , X. Liu , X. Liu , X. Yang , S. Miao , W. Wang , A. Wang , T. Zhang , J. Am. Chem. Soc. 2017, 139, 10790. 28745500 10.1021/jacs.7b05130

[49] G. B.‐C. Marc‐André Légaré , R. D. Dewhurst , E. Welz , I. Krummenacher , B. Engels , H. Braunschweig , Science 2018, 359, 896. 29472479 10.1126/science.aaq1684

[50] C. Ling , X. Niu , Q. Li , A. Du , J. Wang , J. Am. Chem. Soc. 2018, 140, 14161. 30282453 10.1021/jacs.8b07472

[51] G. Shen , R. Zhang , L. Pan , F. Hou , Y. Zhao , Z. Shen , W. Mi , C. Shi , Q. Wang , X. Zhang , J. J. Zou , Angew. Chem., Int. Ed. 2020, 59, 2313. 10.1002/anie.201913080 31743560

[52] Y. Sun , S. Sun , H. Yang , S. Xi , J. Gracia , Z. J. Xu , Adv. Mater. 2020, 32, 2003297. 10.1002/adma.202003297 32776367

[53] Z.‐F. Huang , J. Song , Y. Du , S. Xi , S. Dou , J. M. V. Nsanzimana , C. Wang , Z. J. Xu , X. Wang , Nat. Energy 2019, 4, 329.

[54] C. He , Z.‐Y. Wu , L. Zhao , M. Ming , Y. Zhang , Y. Yi , J.‐S. Hu , ACS Catal. 2019, 9, 7311.

[55] X. Wang , Y. Zhang , H. Si , Q. Zhang , J. Wu , L. Gao , X. Wei , Y. Sun , Q. Liao , Z. Zhang , K. Ammarah , L. Gu , Z. Kang , Y. Zhang , J. Am. Chem. Soc. 2020, 142, 4298. 31999446 10.1021/jacs.9b12113

[56] J. Li , H.‐X. Liu , W. Gou , M. Zhang , Z. Xia , S. Zhang , C.‐R. Chang , Y. Ma , Y. Qu , Energy Environ. Sci. 2019, 12, 2298.

[57] C. Mao , J. Wang , Y. Zou , G. Qi , J. Y. Yang Loh , T. Zhang , M. Xia , J. Xu , F. Deng , M. Ghoussoub , N. P. Kherani , L. Wang , H. Shang , M. Li , J. Li , X. Liu , Z. Ai , G. A. Ozin , J. Zhao , L. Zhang , J. Am. Chem. Soc. 2020, 142, 17403. 32948092 10.1021/jacs.0c06118

[58] W. Peng , M. Luo , X. Xu , K. Jiang , M. Peng , D. Chen , T. S. Chan , Y. Tan , Adv. Energy Mater. 2020, 10, 2001364.

