# Supplementary material for: Boosting Nitrogen Reduction to Ammonia on FeN4 Sites by Atomic Spin Regulation
Source: Adv Sci (Weinh). 2021 Sep 2;8(20):2102915. doi: 10.1002/advs.202102915 (PMC8529464; doi:10.1002/advs.202102915)
Supplement: Supplementary file 1 — Supporting Information [file ADVS-8-2102915-s001.pdf]

WILEY-VCH

© 2021 Wiley-VCH GmbH

ADVANCED SCIENCE
Open Access

Supporting Information

for *Adv. Sci.,* DOI: 10.1002/advs.202102915

Boosting Nitrogen Reduction to Ammonia on FeN_4 sites by Atomic Spin Regulation

Yajin Wang, Wenzheng Cheng, Pengfei Yuan, Gege Yang, Shichun Mu, Jialin Liang, Huicong Xia, Kai Guo, Mengli Liu, Shuyan Zhao, Gan Qu, Bang-An Lu, Yongfeng Hu, Jinsong Hu,* Jia-Nan Zhang**

Supporting Information

Boosting Nitrogen Reduction to Ammonia on FeN_4 sites by Atomic Spin Regulation

Yajin Wang, Wenzheng Cheng, Pengfei Yuan, Gege Yang, Shichun Mu, Jialin Liang, Huicong Xia, Kai Guo, Mengli Liu, Shuyan Zhao, Gan Qu, Bang-An Lu, Yongfeng Hu, Jinsong Hu,* Jia-Nan Zhang**

Ms. Y. Wang, Mr. W. Cheng, Ms. G. Yang, Ms. J. Liang, Mr. H. Xia, Mr. K. Guo, Ms. M. Liu, Ms. S. Zhao, Dr. G. Qu, Dr. B. Lu, Prof. J. Zhang
College of Materials Science and Engineering
Zhengzhou University
Zhengzhou 450001, P. R. China
E-mail: zjn@zzu.edu.cn (J. Zhang)

Prof. J. Hu,
Beijing National Laboratory for Molecular Sciences (BNLMS), CAS Key Laboratory of Molecular Nanostructure and Nanotechnology
Institute of Chemistry, Chinese Academy of Sciences
Beijing 100190, China
E-mail: hujs@iccas.ac.cn (J. Hu)

Prof. S. Mu
State Key Laboratory of Advanced Technology for Materials Synthesis and Processing, Wuhan University of Technology
Wuhan 430070, P. R. China.
Foshan Xianhu Laboratory of the Advanced Energy Science and Technology Guangdong Laboratory, Xianhu hydrogen Valley, Foshan 528200, China

Prof. Y. Hu
Canadian Light Source
44 Innovation Boulevard Saskatoon, SK, S7N 2V3, Canada
E-mail: Hu@lightsource.ca (Y. Hu)

Prof. P. Yuan
International Joint Research Laboratory for Quantum Functional Materials of Henan Province, and School of Physics and Microelectronics, Zhengzhou University, Zhengzhou 450001, P. R. China

Table of Contents

1. Experimental Details

2. Computational Methods and Models

3. Supplementary Figures

4. Supplementary Tables

1. Experimental Section

Chemicals and reagents. Pyromellitic dianhydride and Urea were purchased from Beijing InnoChem Science technology Co., Ltd. Molybdenum (Ⅴ) chloride and Ammonium molybdate (di) were purchased from Macklin. Iron (Ⅲ) chloride anhydrous was bought from Sinopharm Group Chemical Reagent Co., Ltd. Ammonium chloride was obtained from Tianjin Shengao Chemical Reagent Co., Ltd. Nafion (5.0 wt%) was purchased from Sigma-Aldrich. Ketjen Black (KB) was bought from Sinopharm Group Chemical Reagent Co., Ltd. All chemicals were used as received without any further purification. Deionized water was used in all experiments.

Synthesis of FeMoPPc. $FeCl_3$ (16.25 mg, 0.1 mmol), urea (420 mg, 0.007 mol), NH_4Cl (96 mg, 1.8 mmol), $(NH_4)_2Mo_2O_7$ (2.516 mg,0.0074 mmol), $MoCl_5$ （54.7 mg, 0.2 mmol), pyromellitic dianhydride (220 mg, 1 mmol), carbon black (50 mg) were mixed and ground uniformly in an agate mortar for 20 minutes. Then, the mixture was transferred into a crucible, covered with a lid, and placed in a muffle furnace, and heated at 220 °C for 3 hours with a ramp rate of 2 $°C·min^{-1}$. After cooling down to room temperature, the obtained product was washed with deionized water, acetone, and methanol several times. Finally, the product was dried under vacuum at 60 °C for 12 hours to obtain FeMoPPc. The FePPc catalyst was obtained *via* the similar synthetic protocol without the addition of $MoCl_{5.}$ The controlled samples with different metal ratios of $Fe_{0.5}Mo_{0.25}PPc$, $Fe_{0.5}Mo_{0.5}PPc$, $Fe_{0.5}Mo_{0.8}PPc$, $Fe_{0.5}Mo_{1.2}PPc$, $Fe_{0.5}Mo_{1.5}PPc$ were obtained *via* the similar synthetic protocol with 0.05, 0.1, 0.16, 0.24, 0.3 mol of $MoCl_5$ (the amount of $FeCl_3$ remains unchanged), respectively.

Synthesis of MoPPc. $MoCl_5$ (27.35 mg, 0.1 mmol), urea (420 mg, 0.007 mol), NH_4Cl (96 mg, 1.8 mmol), $(NH_4)_2Mo_2O_7$ (2.516 mg,0.0074 mmol), pyromellitic dianhydride (220 mg, 1 mmol), and carbon black (50 mg) were mixed and ground uniformly in an agate mortar for 20 minutes. Then, the mixture was transferred into a crucible, covered with a lid, and placed in a muffle furnace, and heated at 220 °C for 3 hours with a ramp rate of 2 $°C \cdot min^{-1}$. After cooling down to room temperature, the obtained product was washed with deionized water, acetone, and methanol several times. Finally, the product was dried under vacuum at 60 °C for 12 hours to obtain MoPPc.

Characterizations. Transmission electron microscope (TEM, FEI Tecnai G2 20) with an accelerating voltage of 200 kV and field-emission scanning electron microscope (FE-SEM, JEORJSM-6700 F) was employed to observe the morphology of the sample. The HAADF-STEM images were obtained by JEOL JEM-ARM200F at an accelerating voltage of 200 kV. The crystal phases present in each sample were identified using powder X-ray diffraction (XRD) patterns recorded on a Y-2000X-ray Diffractometer with copper Kα radiation (λ=1.5406 Å) at 40 kV, 40 mA. UV/Vis diffuse reflectance spectra was measured by using a U-4100 UV/Vis-NIR spectrometer (Hitachi). The X-ray photoelectron spectroscopy (XPS) measurements were acquired with an ESCA LAB 250 spectrometer on a focused monochromatic Al K_α line (1486.6 eV) X-ray beam with a spot diameter of 200 μm. A micromeritics ASAP 2020 surface area analyzer was used to obtain the N_2 adsorption/desorption curve by BET measurements. Inductively coupled plasma atomic emission spectroscopy (ICP-AES) was used to measure element content on Aglient 5110. The Fe and Mo K-edge X-ray absorption near edge structure (XANES) and the extended X-ray absorption fine structure (EXAFS) were investigated at the SXRMB and Bio-XAS beamlines at the Canadian Light Source. References, such as Fe and Mo foils, are used to

calibrate the beamlie energy and for comparison to samples. Fluorescence detection was performed using a 7-element Si drift detector for samples and the total electron yield was used for measurement of samples with high concentration, such as references. The EXAFS raw data were then background-subtracted, normalized and Fourier transformed by the standard procedures with the IFEFFIT package. A conventional spectrometer (Germany, Wissel MS-500), using a ^{57}Co (Rh) source with activity of 25 mCi, in transmission geometry with constant acceleration mode was used to perform the Mössbauer measurements. The velocity calibration was done with a room temperature α-Fe absorber. The spectra were fitted by the software Recoil using Lorentzian Site Analysis.

Preparation of Working Electrode. 1 mg catalyst were dispersed in 100μL of ethanol and 10μL of Nafion solution to form a homogeneous catalyst ink under sonication for 30min. Then, 50μL of catalyst ink were dropped evenly on carbon paper for catalytic area (1×1 cm^2), and dried at room temperature.

Electrocatalytic measurement. CHI760E electrochemical workstation (CH Instrument Co., Shanghai) was used to perform the electrochemical measurements in a H-type cell separated by a Nafion 115 membrane using a typical three-electrode setup (counter electrode: Pt mesh, 1×1 cm^2; reference electrode: Ag/AgCl, saturated KOH electrolyte). The electrolyte for electrochemical testing was 0.1M KOH solution, 0.1M HCl in absorption bottle was used to absorb NH_3. All potentials were converted to the RHE reference scale by E (*vs* RHE) = E (*vs* Ag/AgCl) + 0.197 V + 0.059 × pH. Before experiment, the electrolyte in the cathode cell was bubbled with pretreated pure N_2 (99.999% purity) for 30 min to eliminate oxygen in solution. Pure N_2 was continuously supplied with a constant gas flow rate in the entire electrolytic process. LSV curves of samples were performed in N_2-saturated and Ar-saturated 0.1

M KOH with the scan rate of 5 mV s^{-1} to examined the electrochemical activities of catalyst. A potentiostatic test was performed in a N_2-saturated 0.1 M KOH solution. After electrolysis, a colorimetry was used to measure ammonia and hydrazine hydrate in the electrolyte and absorber, respectively.

^{15}N isotope labeling experiment. The ^{15}N isotopic labeled experiment were performed using the $^{15}N_2$ isotope to determine the N source of ammonia.[1] First, Ar gas is continuously injected into the electrolyte for 1 hour to remove O_2 and N_2, and then using $^{15}N_2$ as the feeding gas. After electrolysis at -0.3 V (vs. RHE) for 2h in 0.1 M KOH solutions, the resulting electrolyte was concentrated by hearting at 75℃. The analysis of $^{15}NH_3$ product was conducted by the ^{1}H NMR with d^6-DMSO.

Determination of ammonia. The quantitative detection of NH^{4+} in the electrolyte was performed according to the indophenol blue method.[2] After the electrolytic reaction, 2ml of KOH electrolyte was put it in the reaction bottle, and adding 2 mL NaOH (1 M) solution containing 5 wt % salicylic acid and 5 wt % sodium citrate, 1 mL NaClO (0.05 M), and 0.2 mL of 1 wt % $Na[Fe(NO)(CN)_5]$ (sodium nitroferricyanide), respectively. The solutions added into electrolyte were used as color reagent. After 2h of reaction, UV-vis absorbance test was performed at a wavelength of 655nm. The blank electrolyte was used for background determination. The corrected absorbance values of NH_4Cl with different standard concentrations were then measured, and the relationship curve between NH_4^+ ion concentration and absorbance were drawn, which is a calibration curve. Through the calibration curve, the absorbance of the KOH electrolyte after the NRR reaction was been measured. The method of detecting NH^{4+} and the calibration curves of NH_3 in HCl absorber were the same as above. By superimposing the NH_4^+ ion

concentration of KOH electrolyte and HCl absorber, the amount of NH_4^+ ions produced in the NRR can be calculated.

Determination of hydrazine hydrate. The quantitative detection of N_2H_4 in the electrolyte was carried out according to the Watt and Chrisp method.[3] The mixture of para-(dimethylamino) benzaldehyde (5.99 g), HCl (concentrated, 30 mL) and ethanol (300 mL) was used as a color reagent. After the electrolytic reaction, 2 mL of KOH electrolyte was put it in the reaction bottle, and 5 mL above prepared color reagent was added with stirring 15 min at room temperature. The solutions added into electrolyte were used as color reagent. Absorbance test was performed at a wavelength of 460 nm. The blank electrolyte was used for background determination. The corrected absorbance values of N_2H_4 with different standard concentrations were then measured, and the relationship curve between NH_4^+ ion concentration and absorbance were drawn. The remaining steps were the same as the method for detecting ammonia production.

Calculation method for the yield rate of ammonia and faradaic efficiency (FE). The FE of NH_3 production was calculated as follows[2]:

$$FE = N \times V \times C_{NH_4^+} \times F / (Q \times M)$$

N, the number of electrons transferred for product formation, which is 3 for NH_3.

V, the volume of the electrolyte (0.1 M KOH).

$C_{NH_4^+}$, the measured mass concentration of NH_4^+.

F, Faraday constant, 96485.33 C mol^{-1}.

Q, total electric charge.

M: the relative molecular mass of NH_4^+, which is 18 g mol^{-1}

The Yield Rate of NH_3 was calculated as follows:

$$v_{NH_3} = (V \times C_{NH_4^+}) / (m \times t)$$

v_{NH_3}, the yield rate of NH_3.

V, the volume of the electrolyte (0.1 M KOH).

$C_{NH_4^+}$, the measured mass concentration of NH_4^+.

m, the mass of the supported catalyst.

t, the reaction time.

Calculation equation for the number of unpaired d electron (n) of Fe ion.[4]

$$2.828\sqrt{\chi_m T} = \mu_{eff} = \sqrt{n(n+2)}$$

χ_m, magnetic susceptibility.

μ_{eff}, the effective magnetic moment.

n, the number of unpaired d electron.

2. Computational methods and models. First-principles calculations[5] were performed by using the Vienna Ab initio Simulation Package (VASP)[6-10] to investigate the NRR performance of (PPc series). The valence-core electrons interactions were treated by Projector Augmented Wave (PAW)[11,12] potentials and the electron exchange correlation interactions were described by the generalized gradient approximation (GGA) with the Perdew-Burke-Ernzerhof (PBE)[13] functional. Considered long-range interaction at the interface, Van der Waals interactions were considered using DFT-D3 correlation. To avoid interaction originating from other slabs, a vacuum of 20 Å was added along z direction. The convergence criterion of geometry relaxation was set to 0.02 eV•Å^{-1} in force on each atom. The energy

cutoff for plane wave-basis was set to 500 eV. The K points were sampled with 1×1×1 by Monkhorst-Pack method.

Gibbs free energy change (ΔG) was evaluated based on the computational hydrogen electrode (CHE) model, which takes one-half of the chemical potential of gaseous hydrogen under standard conditions as the free energy of the proton-electron pairs. ΔG were calculated by the following equation:

$$\Delta G = \Delta E + \Delta E_{ZPE} - T\Delta S + neU$$

where ΔE, ΔE_{ZPE}, ΔS are the reaction energy from DFT calculation, the correction of zero-point energy and the change of simulated entropy, respectively. T is the temperature (T = 300 K). *n* and U are the number of transferred electrons and applied potential, respectively.

3. Supplementary Figures

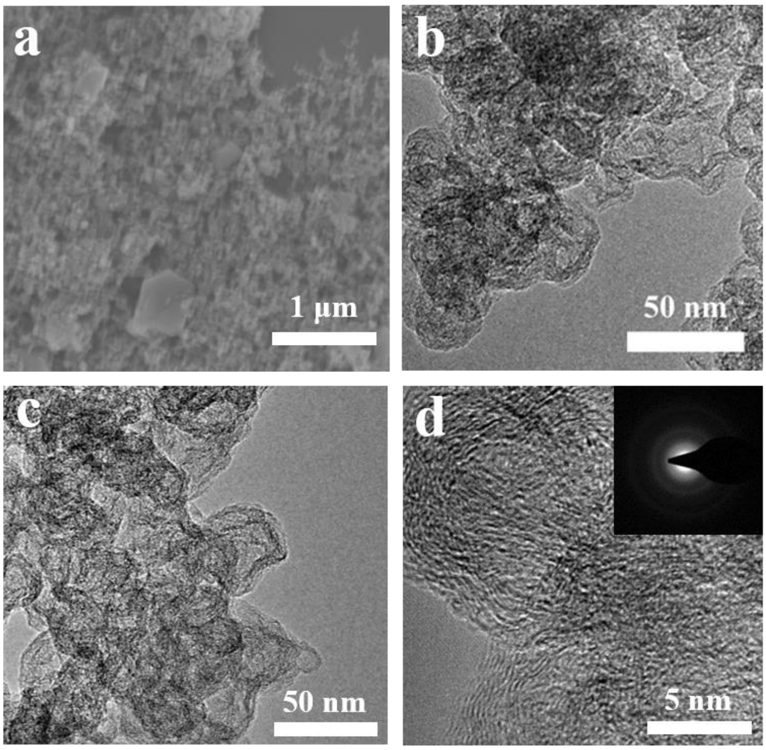

Figure S1. SEM (a), TEM (b, c) and d) HRTEM images of FeMoPPc (inset: selected area electron diffraction image).

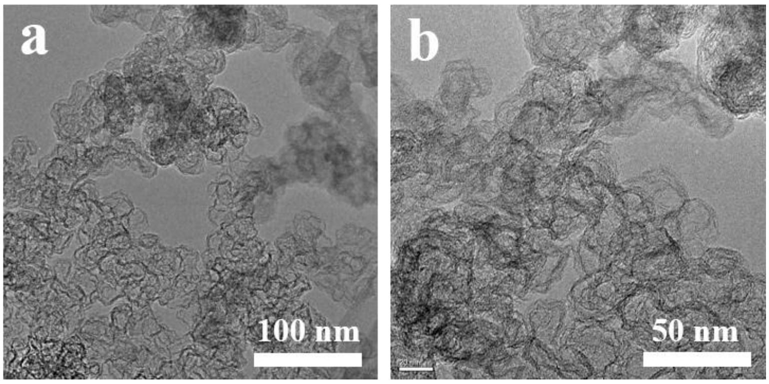

Figure S2. TEM (a, b) images of CB.

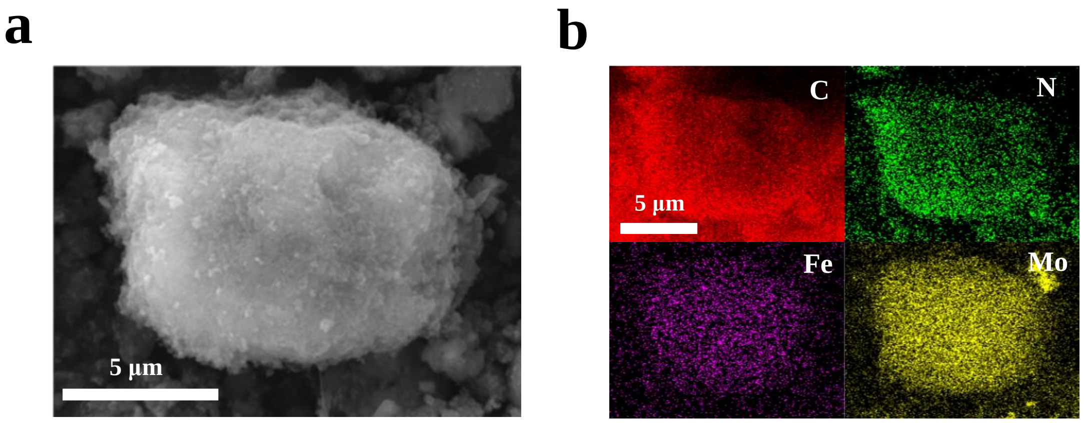

Figure S3. SEM-EDS elemental mapping images of FeMoPPc.

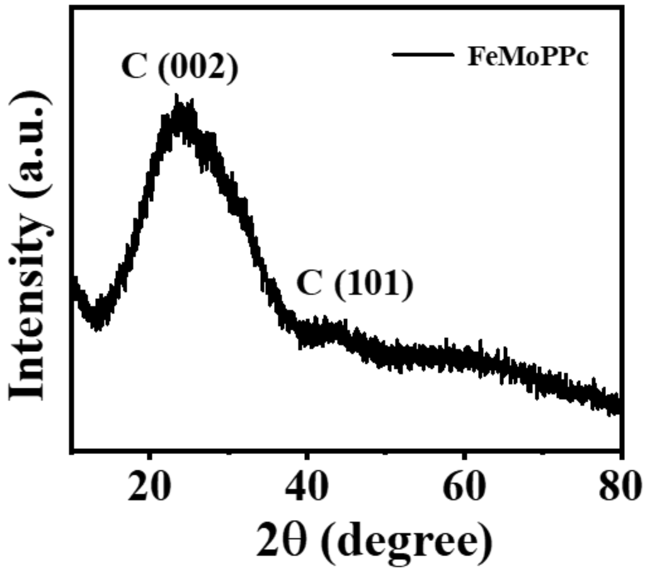

Figure S4. XRD images of FeMoPPc.

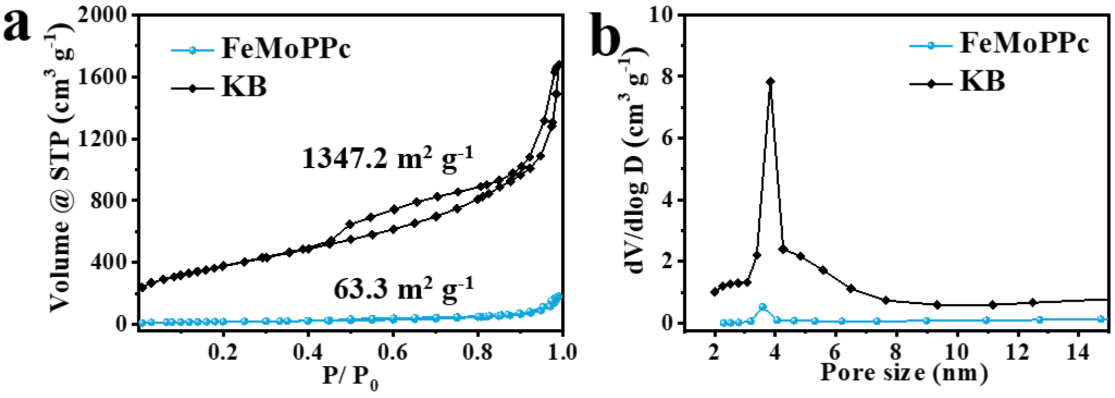

Figure S5. N_2 sorption isotherms (a) and the pore size distribution (b) of KB and FeMoPPc.

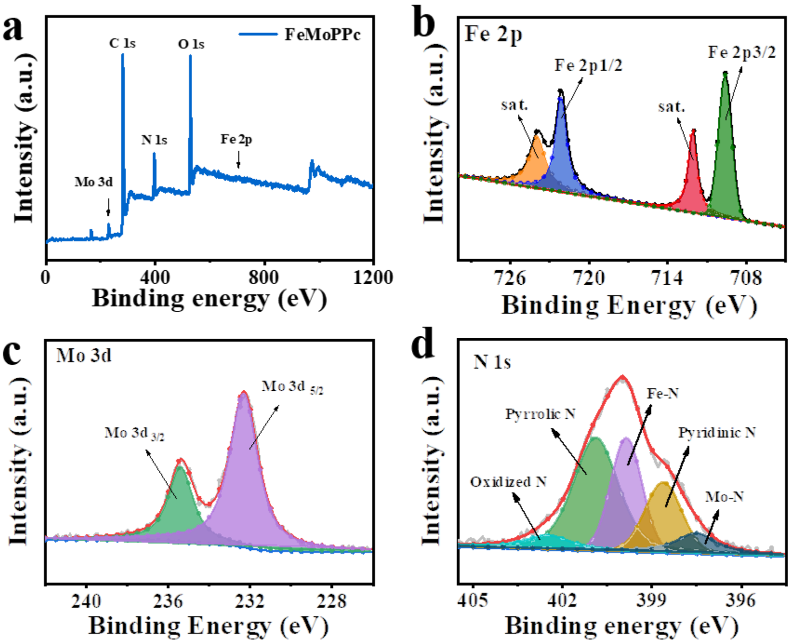

Figure S6. (a) XPS survey spectra, (b) high-resolution Fe 2p, (c) Mo 3d, and (d) N 1s XPS spectra of FeMoPPc.

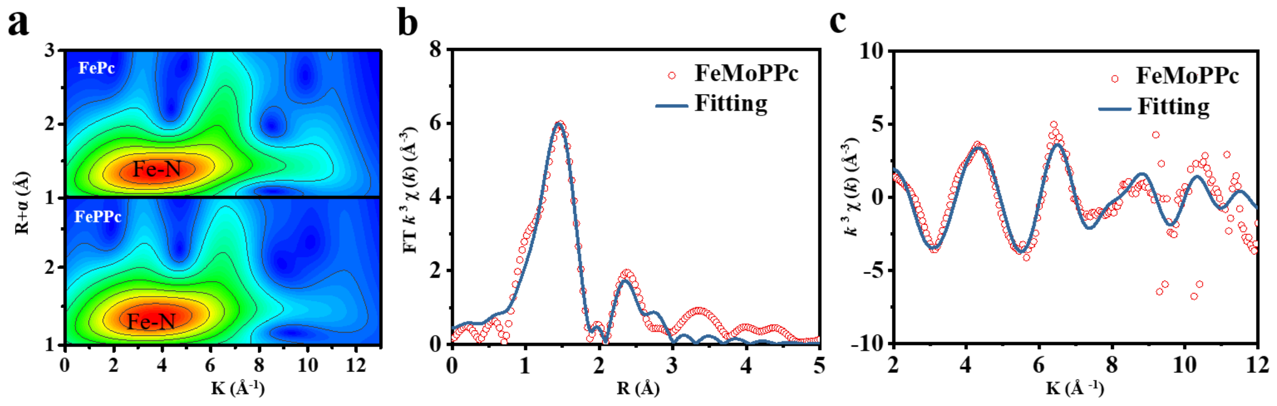

Figure S7. (a) Wavelet transform of the k^3-weighted EXAFS data of FePc, FePPc. (b) Fe K-edge EXAFS R space and (c) k space fitting curve (blue line) and the experimental data (red circles) of the FeMoPPc catalyst.

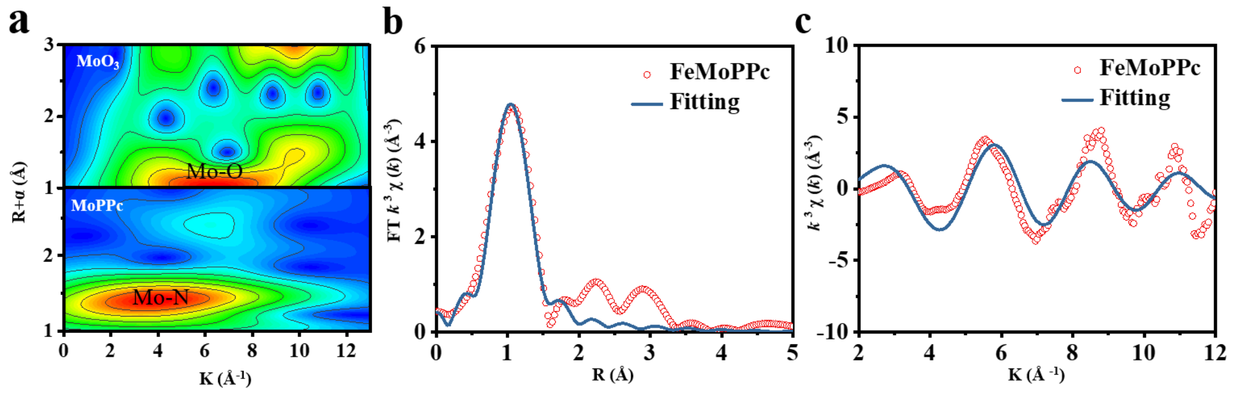

Figure S8. (a) Wavelet transform of the k^3-weighted EXAFS data of MoO_3, MoPPc. (b) Mo K-edge EXAFS R space and (c) K space fitting curve (blue line) and the experimental data (red circles) of the FeMoPPc catalyst.

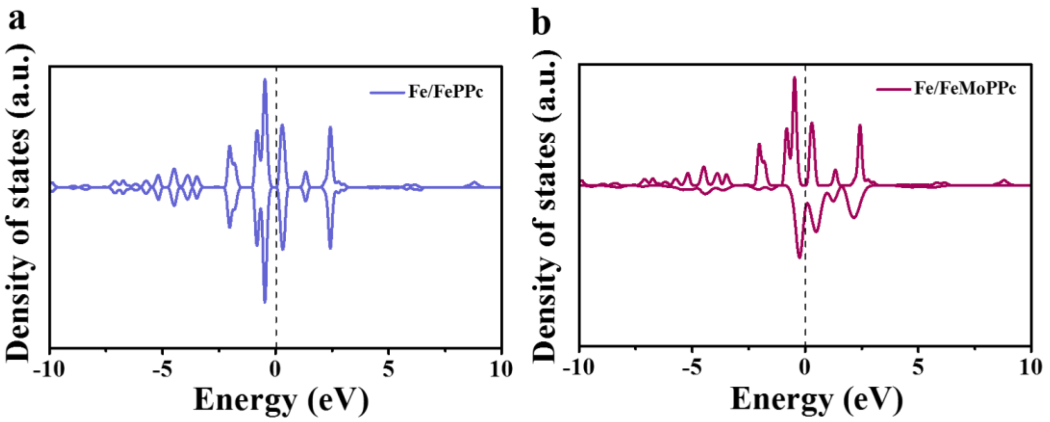

Figure S9. DOS of (a) Fe/FePPc and (b) Fe/FeMoPPc.

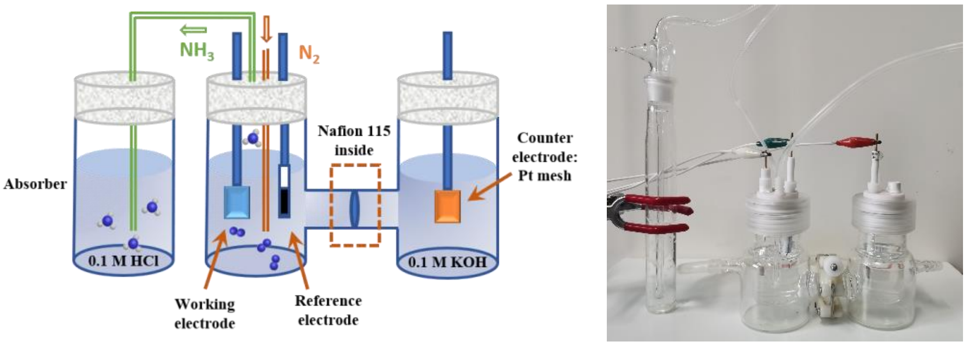

Figure S10. Scheme and photograph of H-type cell for NRR.

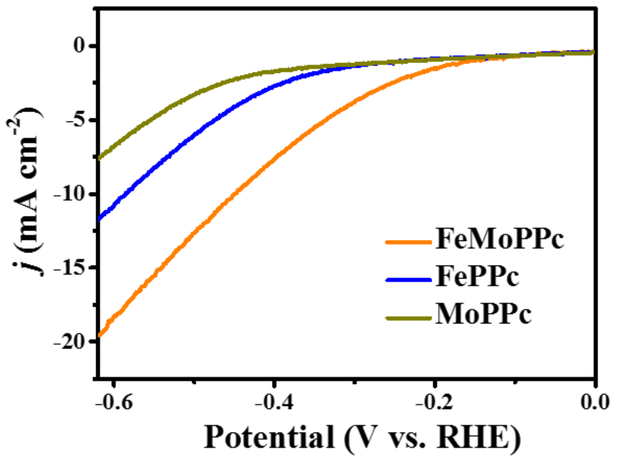

Figure S11. Linear sweep voltammetry curves of FeMoPPc, FePPc, and MoPPc in N_2-saturated 0.1 M KOH solution.

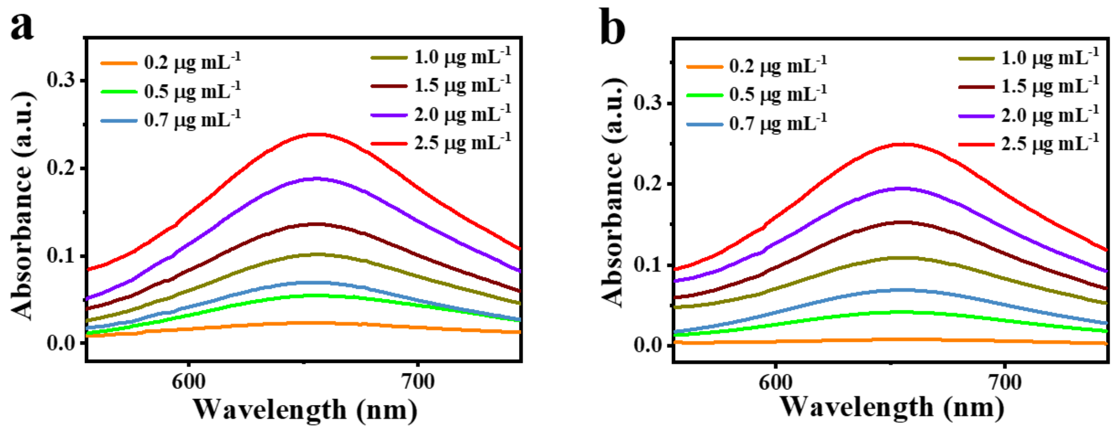

Figure S12. UV-Vis absorption spectrums of NH^{4+} detection. Under alkaline (a) and acidic (b) conditions, absorbance of standard solutions of different concentrations at a wavelength of 655 nm.

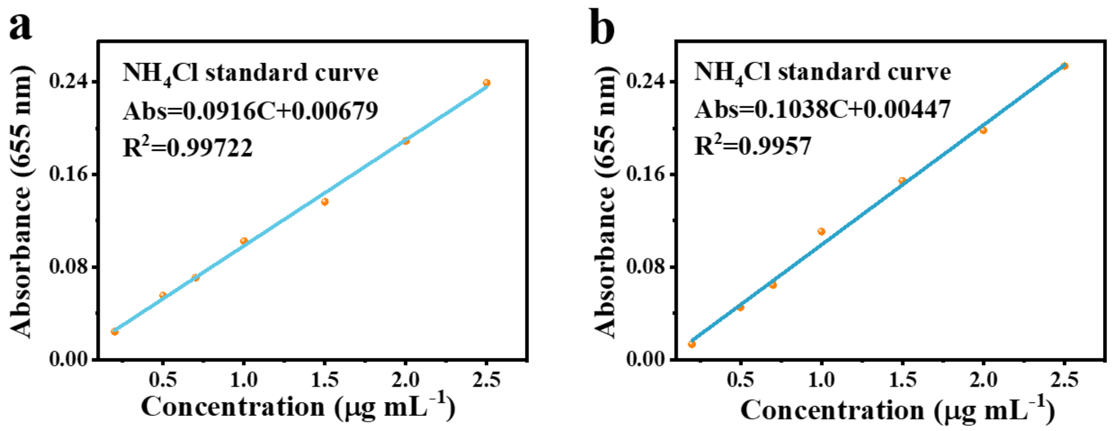

Figure S13. Calibration curve of the indophenol blue method using NH_4Cl solutions of known concentrations as standards at a wavelength 655nm under alkaline (a) and acidic (b) conditions, respectively.

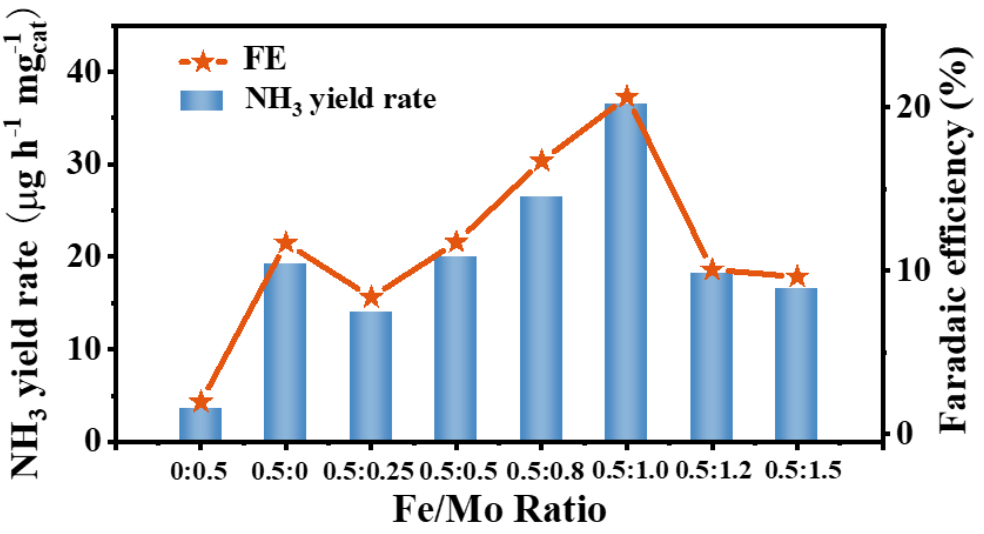

Figure S14. NH_3 yield and Faradaic efficiency with different ratios of Fe and Mo content.

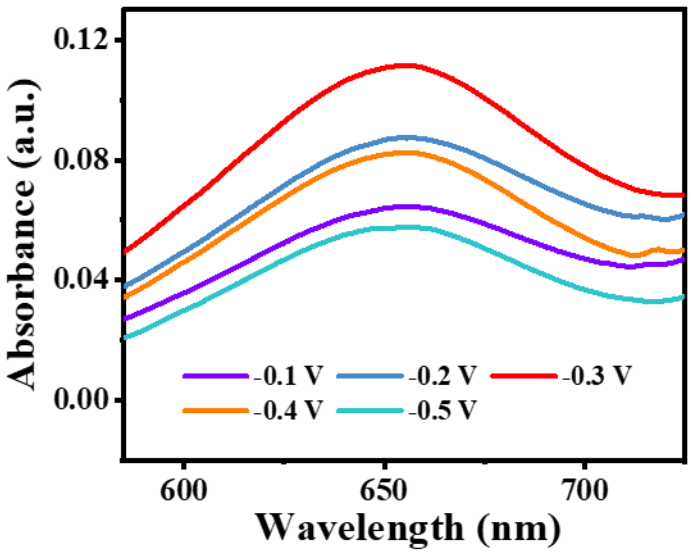

Figure S15. The UV−Vis absorption spectra of after 2 h potentiostatic electrolysis under N_2 atmosphere at different potentials.

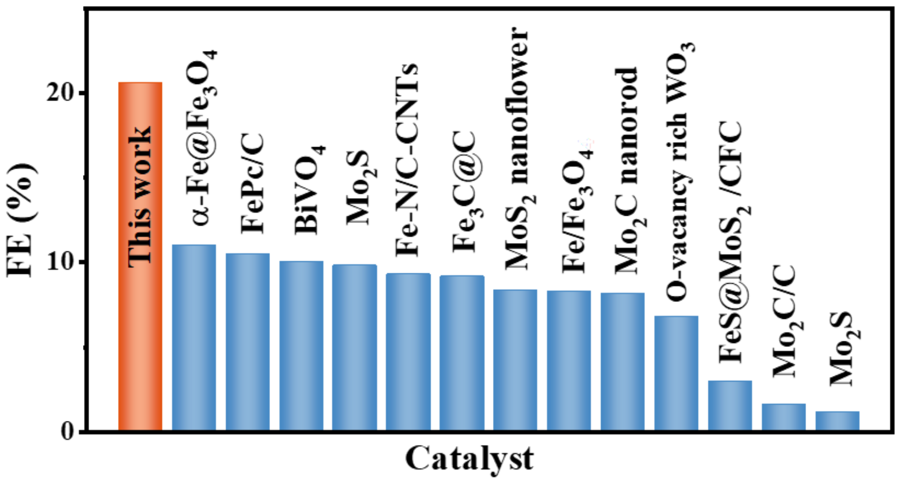

Figure S16. A comparison of the NRR performances of a few representative metallic catalysts for the electroreduction of N_2 to NH_3.

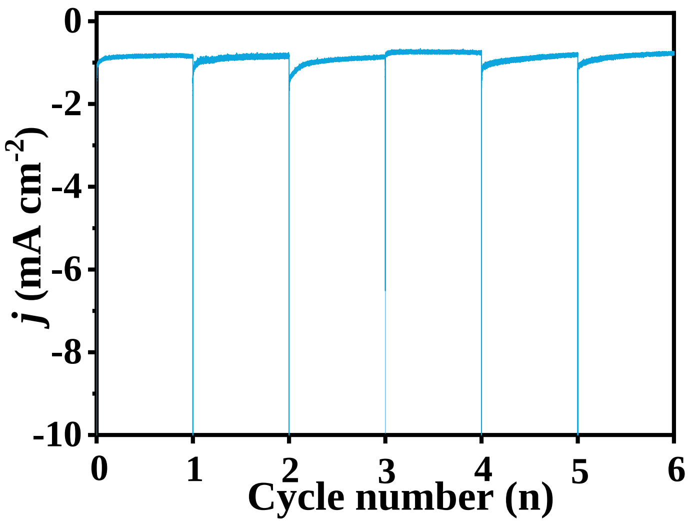

Figure S17. The stability test. Chronoamperometric profile at -0.3 V (vs. RHE) during successive six times recycling tests.

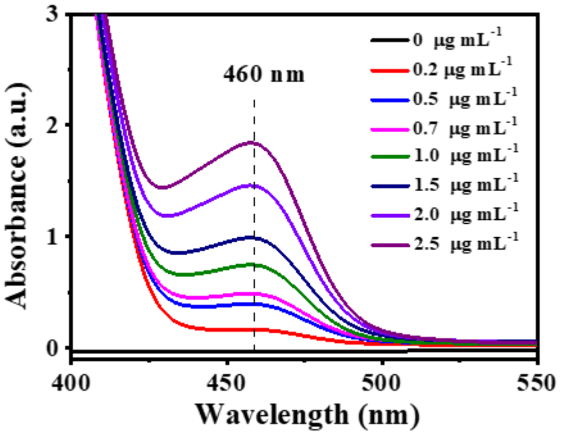

Figure S18. UV-Vis absorption spectrums of $N_2H_4 \cdot H_2O$ detection. Absorbance of N_2H_4 solutions of different concentrations.

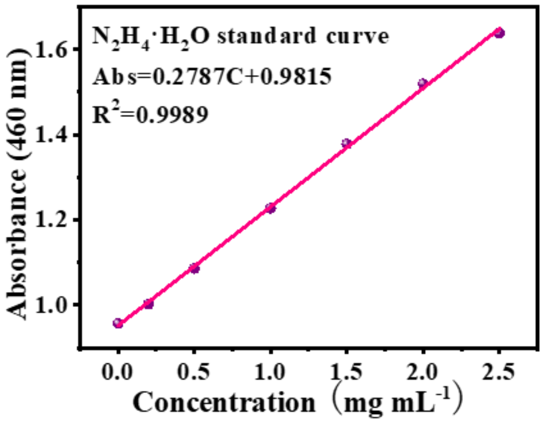

Figure S19. Calibration curve of the watt chrisp method using $N_2H_4{\cdot}H_2O$ solutions of known concentrations as standards at a wavelength 460nm.

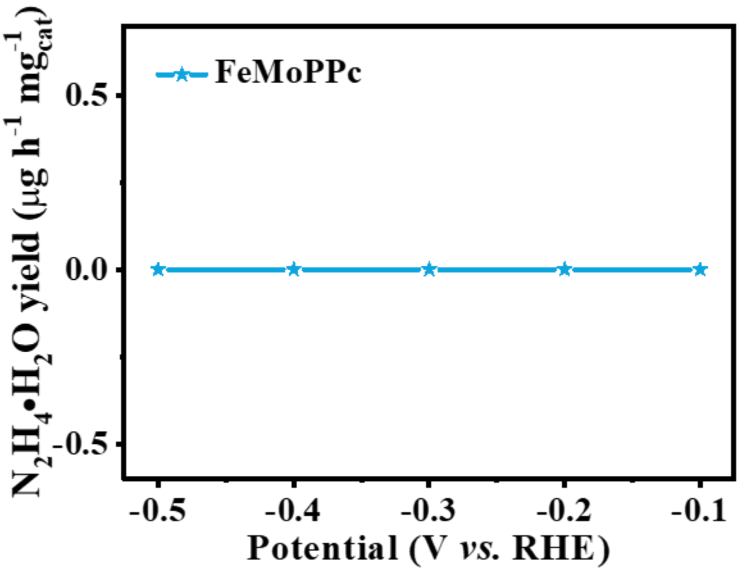

Figure S20. Detection of $N_2H_4{\cdot}H_2O$ yields with FeMoPPc catalyst. There was no $N_2H_4{\cdot}H_2O$ could be detected.

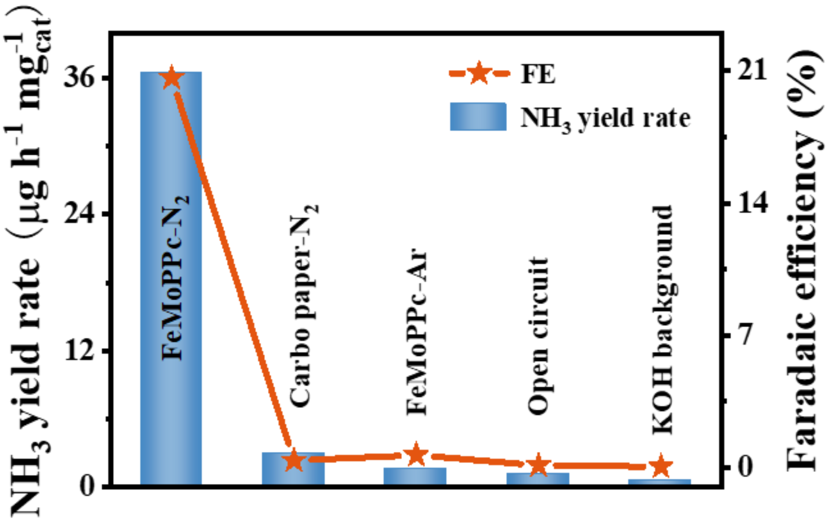

Figure S21. Control experiments using an KOH background, open circuit, a bare carbon paper with the N_2-saturated electrolyte or FePPc@CB with the Ar-saturated electrolyte.

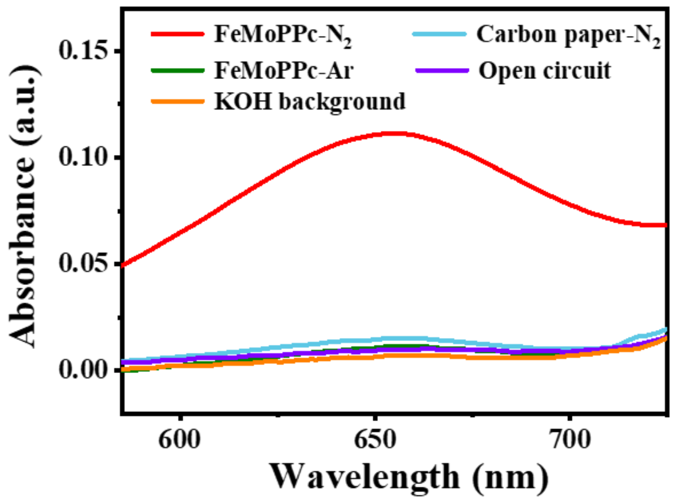

Figure S22. The corresponding UV-Vis absorption spectra of FeMoPPc with the N_2-saturated electrolyte (FeMoPPc-N_2) and control experiments using a bare carbon cloth with the N_2-saturated electrolyte (carbon paper-N_2), FePPc@CB with the Ar-saturated electrolyte (FeMoPPc-Ar), open circuit or a KOH background at -0.3 V (vs. RHE).

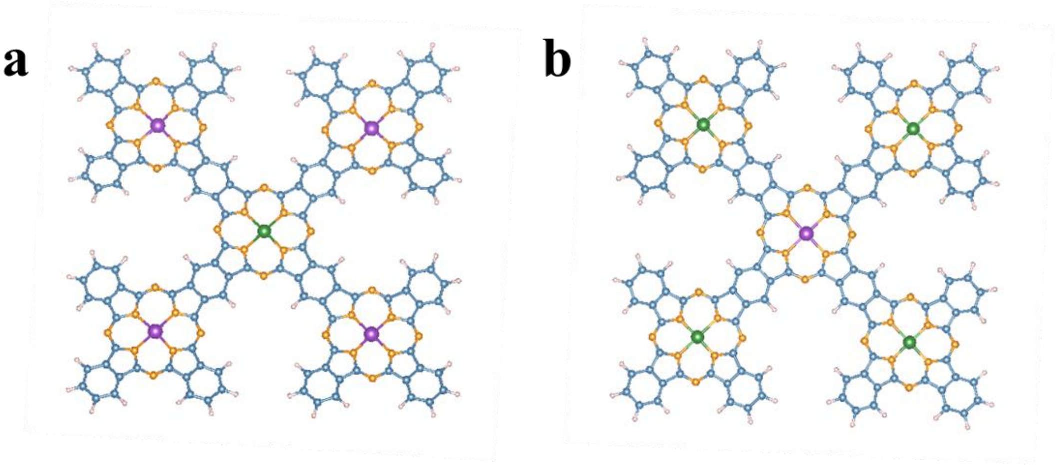

Figure S23. The proposed structure models of $FeMo_4PPc$ (a) and $MoFe_4PPc$ (b).

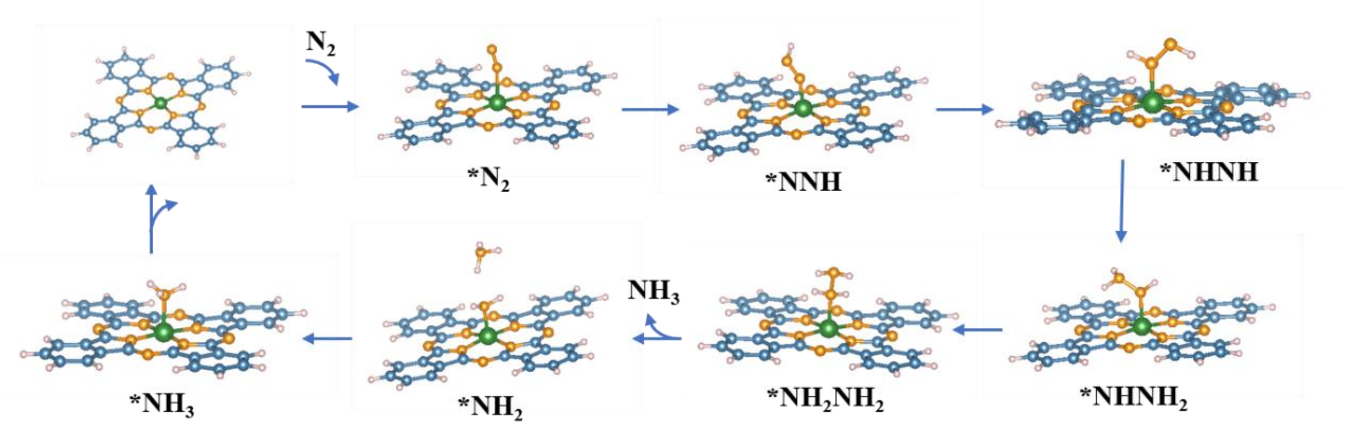

Figure S24. Geometric structures of various intermediates along the alternating pathway of NRR proceeded on FeN_4/FeMoPPc.

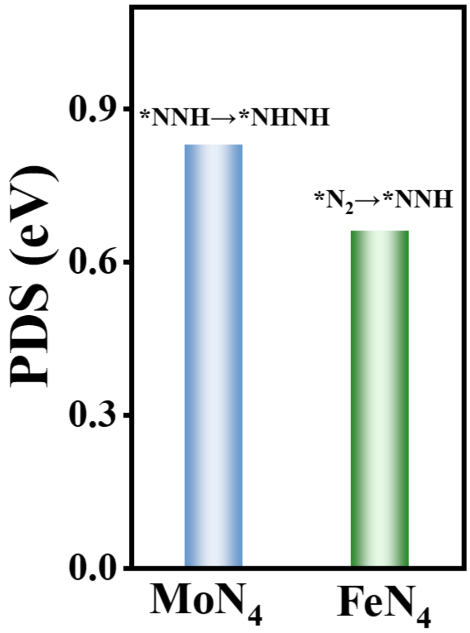

Figure S25. Comparison of PDS on FeN_4/FeMoPPc and MoN_4/FeMoPPc.

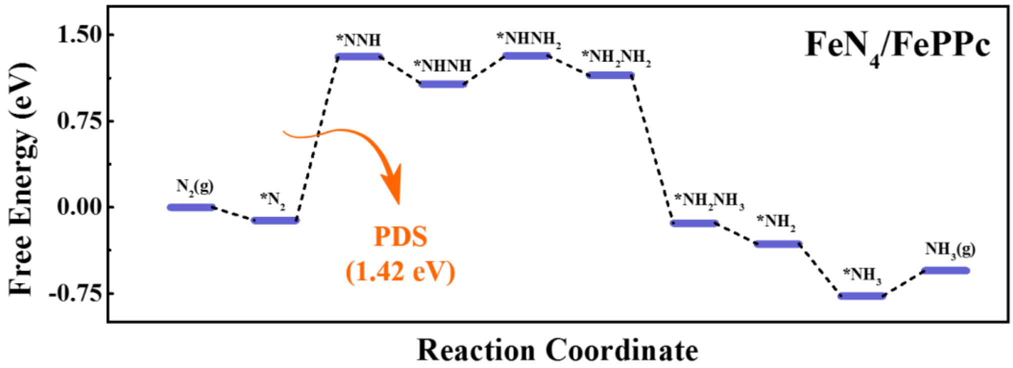

Figure S26. Gibbs free-energy diagrams for the NRR on Fe atom of FePPc.

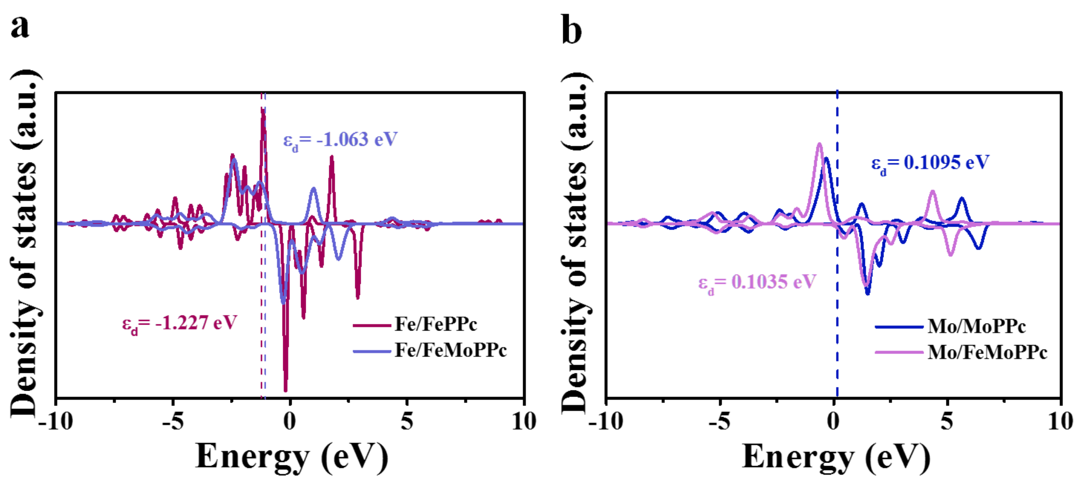

Figure S27. PDOS of Fe active sites (a) and Mo active sites (b).

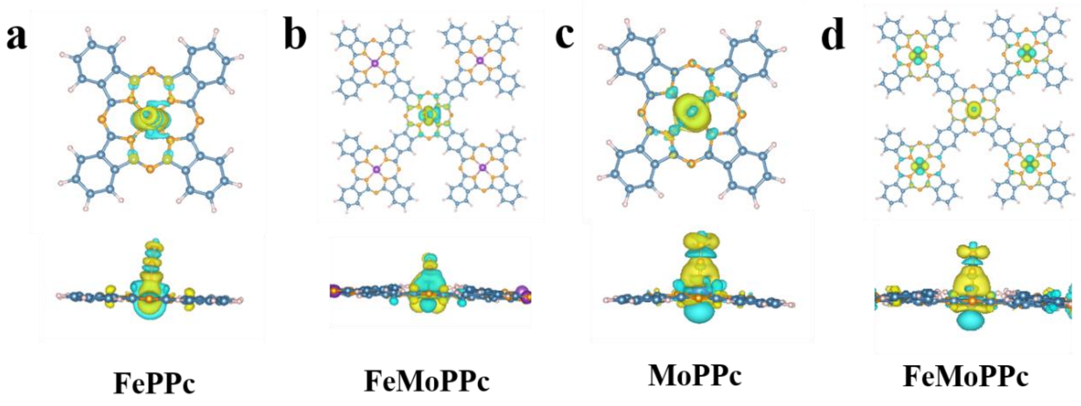

Figure S28. The charge density differences calculations of N_2 bonded to Fe atoms of FePPc (a), FeMoPPc (b). The charge density differences calculations of the N_2 bonded to Mo atoms of MoPPc (c) and FeMoPPc (d). The top is the front view, the bottom is the top view. The electron excess area and electron deficiency area are represented by yellow and blue-green respectively.

4. Supplementary Tables

Table S1. Elements content (wt %) measured by ICP-AES.

Element	Fe	Mo
FeMoPPc	1.28%	5.83%
FePPc	0.7%	--
MoPPc	--	1.53%

Table S2. EXAFS fitting parameters at the Fe and Mo K-edge various samples (S_0^2=0.70 and 0.98).

Sample	Path	C.N.	R (Å)	$\sigma^2\times10^3$ (Å^2)	ΔE (eV)	R factor
**Fe foil**	Fe-Fe	8*	2.47±0.02	4.9±2.0	6.8±3.7	0.002
	Fe-Fe	6*	2.84±0.02	6.0±4.7	5.2±5.9	
**Fe-a**	Fe-N	3.7±0.6	1.96±0.01	11.1±1.8	-2.0±1.8	0.012
	Fe-C	1.1±0.2	2.89±0.03	10.6±4.2	-6.0±5.2	
**Fe-b**	Fe-O	5.8±0.7	1.94±0.01	9.6±1.6	-2.7±1.6	0.010
	Fe-C	2.8±1.7	2.91±0.03	10.5±4.0	-2.0±4.3	
**Mo foil**	Mo-Mo	8*	2.72±0.01	4.2±0.2	-6.2±0.7	0.003
	Mo-Mo	6*	3.15±0.01	4.2±0.3	-2.8±1.1	
**Mo-a**	Mo-O	2.2±1.1	1.73±0.03	6.4±5.6	-8.9±6.7	0.016
**Mo-b**	Mo-N	3.4±0.5	1.76±0.03	8.2±6.2	-9.9±7.9	0.020

Table S3. Summary of the Mössbauer Parameters and Assignments to Different Iron Species in FeMoPPc.

Fe species	Area	Content (%)
Singlet 1	5100	33.7
D1	10100	66.3

Table S4. XPS results analysis for atomic ratio of the prepared samples.

**Fe/Mo**	**0.5:0**	**0.5:0.5**	**0.5:0.7**	**0.5:1.0**	**0.5:1.2**
Atomic ratio	0.22:0	0.22:0.51	0.27:0.64	0.3:0.42	0.2:1.36

Table S5. Comparison of catalytic performance with other electrocatalysts for NRR.[2,14-26]

Catalyst	Electrolyte	Potential (V vs RHE)	FE (%)	Highest NH_3 yield rate	Reference
FeMoPPc	0.1 M KOH	-0.3 V	20.3	33 μg h^{-1} mg^{-1}_{cat}	This work
FePc/C	0.1 M Na_2SO_4	-0.3 V	10.5	10.25 μg h^{-1} mg^{-1}_{cat}	[2]
Mo_2S	0.1M Na_2SO_4	-0.5 V	1.17	4.94 μg h^{-1} cm^{-2}	[14]
Mo_2C/C	0.5 M Li_2SO_4 (pH=2)	-0.2 V	7.8	11.3 μg h^{-1} cm^{-2}	[15]
FeS@MoS_2/CFC	0.1 M Na_2SO_4	-0.5 V	2.96	8.45 μg h^{-1} cm^{-2}	[16]
O-vacancy rich WO_3	0.1 M HCl	-0.12 V	6.8	4.2 μg h^{-1} mg^{-1}_{cat}	[17]
Mo_2C nanorod	0.1 M HCl	-0.3 V	8.13	95.1 μg h^{-1} mg^{-1}_{cat}	[18]
Fe/Fe_3O_4	0.1 M PBS	-0.3 V	8.29	0.19 μg h^{-1} cm^{-2}	[19]
MoS_2 Nanoflower	0.1 M Na_2SO_4	-0.4 V	8.34	11.72 μg h^{-1} cm^{-2}	[20]
Fe_3C@C	0.05 M H_2SO_4	-0.2 V	9.15	8.53 μg h^{-1} mg^{-1}_{cat}	[21]
Fe–N/C–CNTs	0.1 M KOH	-0.2 V	9.28	34.83 μg h^{-1} mg^{-1}_{cat}	[22]
Mo_2S	0.1 M Li_2SO_4 (pH=3.0)	-0.2 V	9.81	26.80 μg h^{-1} cm^{-2}	[23]
$BiVO_4$	0.2 M Na_2SO_4	-0.5 V	10.04	8.60 μg h^{-1} mg^{-1}_{cat}	[24]
SA-Mo/NPC	0.1 M KOH	-0.3 V	14.6	34.0 μg h^{-1} mg^{-1}_{cat}	[25]
0.57Mn_3O_4/b-TiO_2	0.1 M KOH	-0.45 V	25.2	9.85 μg h^{-1} cm^{-2}	[26]

Table S6. Calculated the values of zero-point energy (ΔE_{ZPE}) and entropy change (ΔS) of different adsorption species, where the * denotes the adsorption site. T was set to 300K.

**Adsorption Species**	E_{ZPE} **(eV)**	TS **(eV)**
**$*N_2$**	0.18	0.23
***NNH**	0.43	0.19
***NHNH**	0.81	0.15
**$*NHNH_2$**	1.15	0.13
**$*NH_2NH_2$**	1.26	0.17
**$*NH_2NH_3$**	1.71	0.24
**$*NH_2$**	0.63	0.15
**$*NH_3$**	0.89	0.08

References

[1] M. Wang, S. Liu, T. Qian, J. Liu, J. Zhou, H. Ji, J. Xiong, J. Zhong, C. Yan, *Nat. Commun.* **2019**, 10, 341.

[2] C. He, Z.-Y. Wu, L. Zhao, M. Ming, Y. Zhang, Y. Yi, J.-S. Hu, *ACS Catal.* **2019**, 9, 7311.

[3] Z. H. Xue, S. N. Zhang, Y. X. Lin, H. Su, G. Y. Zhai, J. T. Han, Q. Y. Yu, X. H. Li, M. Antonietti, J. S. Chen, *J. Am. Chem. Soc.* **2019**, 141, 14976.

[4] Z. Li, Z. Zhuang, F. Lv, H. Zhu, L. Zhou, M. Luo, J. Zhu, Z. Lang, S. Feng, W. Chen, L. Mai, S. Guo, *Adv. Mater.* **2018**, 30, 1803220.

[5] W. Kohn, L. J. Sham, *Phys. Rev.* **1965**, 140, A1133.

[6] G. Kresse, J. Hafner, *Phys. Rev. B* **1994**, 49, 14251.

[7] G. Kresse, J. Furthmu¨ller, *Phys. Rev. B* **1996**, 54, 169.

[8] G. Kresse, J. Hafner, *Phys. Rev. B* **1993**, 47, 558.

[9] J. P. Perdew, Y. Wang, *Phys. Rev. B* **1992**, 45, 13244.

[10] G. Kresse, J. Furthmüller, *Comput. Mater. Sci.* 1996, 6, 15.

[11] P. E. Blöchl, *Phys. Rev. B* **1994**, 50, 17953.

[12] G. Kresse, D. Joubert, *Phys. Rev. B* 1999, 59, 1758.

[13] J. P. Perdew, K. Burke, M. Ernzerhof, *Phys. Rev. Lett.* 1996, 77, 3865.

[14] L. Zhang, X. Ji, X. Ren, Y. Ma, X. Shi, Z. Tian, A. M. Asiri, L. Chen, B. Tang, X. Sun, *Adv. Mater.* **2018**, 30, 1800191.

[15] H. Cheng, L. X. Ding, G. F. Chen, L. Zhang, J. Xue, H. Wang, *Adv. Mater.* **2018**, 30, 1803694.

[16] Y. Guo, Z. Yao, B. J. J. Timmer, X. Sheng, L. Fan, Y. Li, F. Zhang, L. Sun, *Nano Energy* **2019**, 62, 282.

[17] Z. Sun, R. Huo, C. Choi, S. Hong, T.-S. Wu, J. Qiu, C. Yan, Z. Han, Y. Liu, Y.-L. Soo, Y. Jung, *Nano Energy* **2019**, 62, 869.

[18] X. Ren, J. Zhao, Q. Wei, Y. Ma, H. Guo, Q. Liu, Y. Wang, G. Cui, A. M. Asiri, B. Li, B. Tang, X. Sun, *ACS Cent. Sci.* **2019**, 5, 116.

[19] L. Hu, A. Khaniya, J. Wang, G. Chen, W. E. Kaden, X. Feng, *ACS Catal.* **2018**, 8, 9312.

[20] X. Li, T. Li, Y. Ma, Q. Wei, W. Qiu, H. Guo, X. Shi, P. Zhang, A. M. Asiri, L. Chen, B. Tang, X. Sun, *Adv. Energy Mater.* **2018**, 8, 1801357.

[21] M. Peng, Y. Qiao, M. Luo, M. Wang, S. Chu, Y. Zhao, P. Liu, J. Liu, Y. Tan, *ACS Appl. Mater. Interfaces* **2019**, 11, 40062.

[22] Y. Wang, X. Cui, J. Zhao, G. Jia, L. Gu, Q. Zhang, L. Meng, Z. Shi, L. Zheng, C. Wang, Z. Zhang, W. Zheng, *ACS Catal.* **2018**, 9, 336.

[23] Y. Liu, M. Han, Q. Xiong, S. Zhang, C. Zhao, W. Gong, G. Wang, H. Zhang, H. Zhao, *Adv. Energy Mater.* **2019**, 9, 1803935.

[24] J. X. Yao, D. Bao, Q. Zhang, M. M. Shi, Y. Wang, R. Gao, J. M. Yan, Q. Jiang, *Small Methods* **2018**, 3, 1800333.

[25] L. Han, X. Liu, J. Chen, R. Lin, H. Liu, F. Lu, S. Bak, Z. Liang, S. Zhao, E. Stavitski, J. Luo, R. R. Adzic, H. L. Xin, *Angew. Chem. Int. Ed.* **2019**, 58, 2321.

[26] J. Zhang, Y. Tian, T. Zhang, Z. Li, X. She, Y. Wu, Y. Wang, J. Wu, *ChemCatChem* **2020**, 12, 2760.
